# Extension of an Atom–Atom Dispersion Function to Halogen Bonds and Its Use for Rational Design of Drugs and Biocatalysts

**DOI:** 10.1021/acs.jpca.0c11347

**Published:** 2021-02-23

**Authors:** Wiktoria Jedwabny, Edyta Dyguda-Kazimierowicz, Katarzyna Pernal, Krzysztof Szalewicz, Konrad Patkowski

**Affiliations:** † Department of Chemistry, Wrocław University of Science and Technology, Wybrzeże Wyspiańskiego 27, 50-370 Wrocław, Poland; ‡ Institute of Physics, Łódź University of Technology, Wólczańska 219, 90-924 Łódź, Poland; § Department of Physics and Astronomy, University of Delaware, Newark, Delaware 19716, United States; ∥ Department of Chemistry and Biochemistry, Auburn University, Auburn, Alabama 36849, United States

## Abstract

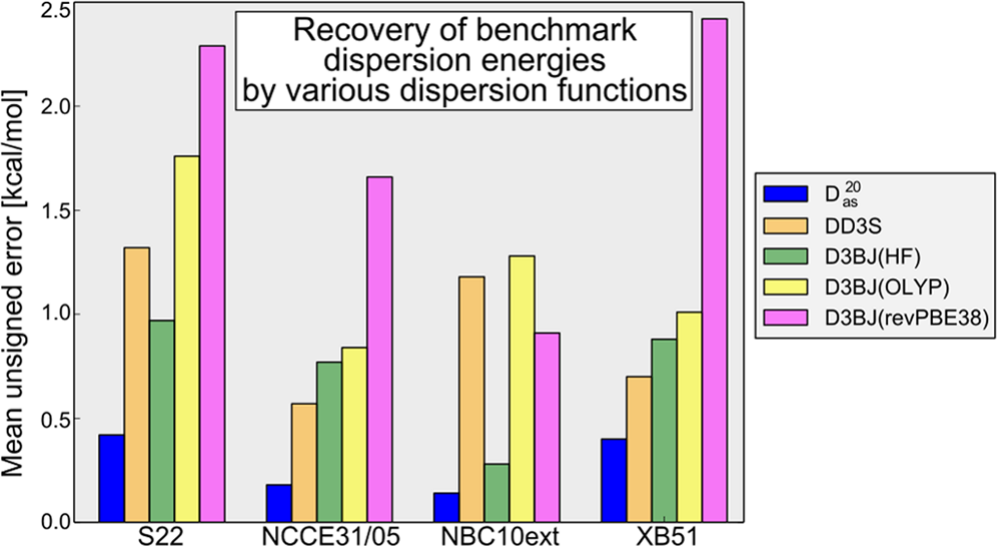

A dispersion function *D*_as_ in the form of a damped atom–atom asymptotic expansion fitted to *ab initio* dispersion energies from symmetry-adapted perturbation theory was improved and extended to systems containing heavier halogen atoms. To illustrate its performance, the revised *D*_as_ function was implemented in the multipole first-order electrostatic and second-order dispersion (MED) scoring model. The extension has allowed applications to a much larger set of biocomplexes than it was possible with the original *D*_as_. A reasonable correlation between MED and experimentally determined inhibitory activities was achieved in a number of test cases, including structures featuring nonphysically shortened intermonomer distances, which constitute a particular challenge for binding strength predictions. Since the MED model is also computationally efficient, it can be used for reliable and rapid assessment of the ligand affinity or multidimensional scanning of amino acid side-chain conformations in the process of rational design of novel drugs or biocatalysts.

## 1 Introduction

The rational drug or material design process would greatly benefit from the availability of rapid nonempirical estimates of relative stabilities of numerous possible conformations of interacting subunits. Due to the large size of molecular systems involved, the currently available state-of-the-art techniques like symmetry-adapted perturbation theory (SAPT)^1^ would be too costly to deal with such a task. On the other hand, preliminary tests involving hydrogen-bonded dimers,^2^ several groups of protein–inhibitor systems,^3−6^ and ionic liquids^7^ indicate that rankings of relative stabilities could be well represented by a scoring model consisting of multipole electrostatic and dispersion terms alone^3^ (MED = *E*_EL,MTP_^(10)^ + *D*_as_), where *E*_EL,MTP_^(10)^ and *D*_as_ denote, respectively, the electrostatic energy in the multipole approximation and the dispersion energy. Importantly, such rankings are quite insensitive to the usage of geometries far from equilibrium,^3,8,9^ which can be beneficial for the *in silico* drug design process where distances between ligands and protein binding sites are frequently inaccurate.

Whereas the long-range electrostatic multipole term could be rapidly estimated from any multicenter multipole expansion, accurate calculations of the dispersion term for large dimers, even in the asymptotic form, are extremely costly. This problem has been circumvented by an application of atom–atom dispersion functions (*D*_as_) developed by a fitting of accurate values of dispersion and exchange-dispersion energies obtained, for a training set of complexes,^10,11^ using SAPT based on density functional theory [SAPT(DFT)]. Due to the importance of drugs containing halogens that were only partially represented in the original *D*_as_ formulation, in this work, we decided to extend the *D*_as_ training set by additional dimers containing such atoms. In particular, bromine- and iodine-containing halogen-bonded complexes from the X40 database^12^ were included in the *D*_as_ training set. Moreover, dimers, including several other new (relative to the previous version of *D*_as_ from ref (11), which will be denoted as *D*_as_^10^) atoms: B, Al, Si, and P, were added. Subsequently, all parameters were refitted.

The earlier versions of *D*_as_ have been used in several research projects, in particular in investigations of protein–ligand complexes. In the first attempt at ligand scoring with such a simple MED model, the inhibitory potency of 22 inhibitors of fatty acid amide hydrolase (FAAH) was estimated.^3^ The first available *D*_as_ version^10^ (*D*_as_^09^) was used in this MED model. Then, the MED model with the *D*_as_^10^ version^11^ was applied in ref (6) to *Trypanosoma brucei* pteridine reductase 1 (*Tb*PTR1) inhibitors, as well as to the inhibitors of the following protein–protein interactions: erythropoietin producing hepatocellular carcinoma A2 receptor-ephrin A1 (EphA2–ephA1, ref (4)) and menin-mixed lineage leukemia protein (menin–MLL, refs (13) and (5)) complexes. In the latter system, two separate studies on different inhibitor classes were performed, namely, thienopyrimidines (in the present contribution referred to as menin–MLL (I))^13^ and a series of subsequently developed ligands bearing a modified thienopyrimidine scaffold (menin–MLL (II)).^5^ Recently, the MED model with the *D*_as_ parameters from the present work (*D*_as_^20^) was applied to five halogen-bonded inhibitors of phosphodiesterase 5 (PDE5), as described in ref (14). Considering the performance of MED in comparison with the various scoring approaches, essentially all of the results obtained in our studies of protein–inhibitor complexes support the conclusion about the favorable MED performance over a number of routinely used scoring functions.^3−6,13,14^ For all of these receptor–ligand systems, the MED model consistently yielded the ranking of inhibitors on par with the best empirical scoring functions, with no more than two such functions providing slightly better results and a dozen or so functions performing worse. In particular, in the case of menin–MLL (I) complexes, the MED-derived scoring outperformed 14 empirical scoring approaches, yielding the best estimate of the experimental binding potency.^13^ What should be emphasized is that no consistency among the top empirical scoring functions was obtained, as some functions featuring the best performance for a particular protein–inhibitor system performed rather poorly for other systems.^4^ It seems that the scoring approaches that rely on empirical parameters derived with arbitrarily selected datasets, might not be general enough to be applicable to all receptor–ligand systems. On the other hand, the nonempirical character of the MED model, associated with the lack of calibration or training on experimentally determined affinity data, renders it valid for a vast repertoire of complexes.

In addition to protein–ligand scoring, a research area that could possibly benefit from the accessibility of a reliable, low-cost binding energy estimate is the de novo enzyme design. The computational development of novel enzymes featuring a predetermined catalytic activity is a rapidly evolving field.^15,16^ Despite a number of successful designs,^17,18^ the catalytic properties of artificial biocatalysts remain within the range attainable by catalytic antibodies, falling behind the outstanding efficiency of naturally evolved enzymes.^19^ Clearly, further advances in the design methodology are required, including the increased precision of designed structures and an improved description of protein–reactant interactions. The catalytic activity of enzymes arising from the lowering of the free-energy barrier of the catalyzed reaction is mainly determined by the magnitude of the transition state stabilization relative to the substrate binding. According to the differential transition state stabilization (DTSS) approach,^20,21^ the stronger a given molecular environment binds the transition state compared to the substrate, the lower is the corresponding activation energy barrier and the faster the catalyzed reaction proceeds. In the case of a multistep reaction, the most significant differential stabilization might, in principle, accompany the rate-limiting step, i.e., the one associated with the highest activation energy.^22^ As demonstrated by Beker et al.^23^ for the reaction catalyzed by ketosteroid isomerase (KSI), the concept of differential stabilization could also be applied to the reaction intermediate that experiences the strongest binding by the enzyme-active site. Differential intermediate state stabilization (DISS) is then expressed in terms of the enzyme–intermediate and enzyme–substrate interaction energy, which in turn can be calculated at various levels of theory, depending on the expected accuracy and computational affordability. As pointed out in ref (24), reliable modeling of enzymatic reactions should account for the dispersion effects. The significance of dispersion interactions for catalytic effects has recently been further reinforced by demonstrating their role in enantioselective reactions.^25^ Therefore, the application of the MED model could bring the low computational cost and the robustness of a nonempirical binding energy evaluation into the *in silico* estimation of enzyme catalytic efficiency.^23^ MED has already been applied to study enzymatic catalysis in ref (23). For a series of KSI mutants, it was shown that the DISS values of two amino acid residues undergoing mutation, obtained with the *E*_EL,MTP_^(10)^ + *D*_as_ model, correlate with the experimental catalytic activity of the particular KSI variants, validating the relevance of the MED model for catalytic efficiency prediction.

Another aspect of biocatalysis where the knowledge of the dispersion energy is critical is the effect of amino acid side-chain rotamers. The importance of accounting for such rotamers has recently been emphasized in ref (26). As demonstrated by Beker and Sokalski,^27^ the vast combinatorial space of side-chain rotamers could be screened by employing atomic multipole representation of the interaction energy, with the purpose of establishing the optimal amino acid conformations constituting the preorganized active site environment. Considering the importance of dispersion interactions for the proper description of the enzyme structure and function, supplementing the rotamer scanning methodology by *D*_as_ term would extend the applicability of this approach to enzymes featuring significant dispersion contribution.

Overall, the purpose of this work is to develop, test, and determine the applicability of the revised *D*_as_ function, in particular in the context of the associated MED model. Demonstrating favorable performance of the *D*_as_ function and the MED model for the description of intermolecular interactions, including those related to enzyme inhibition and biocatalysis, should be of particular interest to the fields of in silico drug and enzyme design.

In the present paper, we describe the development of the new *D*_as_ function (Section 2) and evaluate its performance with respect to benchmark results for small dimers from the training set (Section 3.1) and test sets (Section 3.2). In Section 3.3, the five inhibitors of the urokinase-type plasminogen activator (uPA),^28^ as well as the seven alcohol dimers studied by Hoja et al.,^2^ are examined with the revised MED model. The obtained results are discussed and compared with previously studied protein–ligand systems.^3−6,13,14^ The uPA inhibitors have been deliberately selected for testing the MED approach, as they belong to one of the rare documented cases in the literature^9,28^ where the inhibitor-binding site short contacts resulting from the use of conventional force field optimization have been compared to *ab initio* MP2 results. Such structures constitute a particular challenge for the assessment of binding energy, as both high-level quantum chemistry methods and empirical scoring functions perform rather poorly in terms of predicting the relative stability.^3^ The choice of hydrogen-bonded alcohol dimers emerged from the unusually high contribution of the dispersion interactions,^2^ not commonly seen in the case of hydrogen bonding. The significance of dispersion contribution in this particular case prompted us to test the MED capability of yielding a reliable estimate of the relative stability. In Section 3.4, we apply the MED model to the description of the catalytic contribution of KSI active site residues. In particular, we aim at the determination of the most catalytically active KSI residues and assessment of the role of dispersive interactions in the total DISS characterizing the KSI-catalyzed reaction. To validate the relevance of the rotamer scanning approach to enzymes with a significant share of dispersion interactions, we attempt to determine the KSI side-chain conformations optimal for catalysis by a combination of the MED model with a methodology that enables scanning of the rotamer library.^27^

## 2 Computational Methods

### 2.1 Electrostatic Multipole Expansion

We start with a brief description of the electrostatic component, which will be used in the MED model. The first-order electrostatic multipole term, *E*_EL,MTP_^(10)^, represents the interaction of permanent multipole moments of two isolated monomers. Monomers’ center of mass (COM) moments can be partitioned into atom-centered or bond segment-centered moments, yielding much better multipole expansion convergence at short intermolecular distances compared to the COM expansion. Such distributed moments constitute a natural extension of Mulliken’s population analysis, and the inclusion of higher moments significantly improves properties like molecular electrostatic potentials, electric fields, or multipole electrostatic interaction energies in comparison to the analogous properties derived from monopole moments only.^29−31^ The multipole expansion can be written as
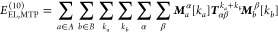
1where ***M***_*a*_^α^[*k*_*a*_] and ***M***_*b*_^β^[*k*_*b*_] are α and β components of atom-centered multipole tensors of rank *k*_*a*_ and *k*_*b*_ for interacting molecules A and B, respectively, and ***T***_αβ_^*k*_*a*_+*k*_*b*_^ is the αβ element of the Cartesian interaction tensor containing the partial derivatives of |***R***_*ab*_|^–1^ of rank *k*_*a*_ + *k*_*b*_. The zero in the superscript indicates that the multipole moments are calculated from Hartree–Fock (HF) densities. We employ here exponent-truncated series, including all terms up to *R*^–*n*^, which converges much better than moment-truncated series terminated at a certain highest multipole moment.^32^ Cumulative atomic multipole moments (CAMM) reported herein for uPA inhibitors and alcohol dimers (Section 3.3) have been calculated at the HF level of theory using a modified^33^ GAMESS^34^ program (available as an IAMM option in the ELMOM section since version 2014/R1). The modeling of biocatalyst interactions has been facilitated by the CAMM library^35^ for amino acid side-chain rotamers,^36^ generated using the HF method and the 6-31G(d)^37−39^ basis set. Unless otherwise stated, the atomic multipole expansion was truncated at the *R*^–5^ term and the 6-31G(d) basis set was used.

### 2.2 Dispersion Energy Expression

The *D*_as_ function,^10,11^ expressed as the sum of atom–atom contributions, allows accounting for the dispersion energy missing in the HF approach and in semilocal DFT functionals. This function has the following form
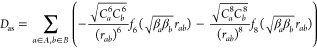
2where *a* and *b* denote atoms in monomers *A* and *B*, respectively, and *f*_*n*_(*r*) is the Tang–Toennies^40^ damping function
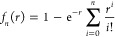
3The parameters *C*_*x*_^*n*^ and β_*x*_, *x* = *a*, *b* are fitted to the sum of the SAPT(DFT)^41−49^ dispersion (*E*_disp_^(2)^) and exchange-dispersion (*E*_exch-disp_^(2)^) energy values, *E*_dispx_^(2)^
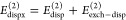
4The parameters were nonlinearly optimized using the functional
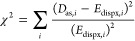
5where the sum extends over all geometries of all dimers. The values of the parameters were constrained to be positive during the optimization. The starting values of the parameters already present in *D*_as_^10^ were taken from that function. The new parameters were selected randomly within arbitrarily chosen ranges. First, we have used a genetic algorithm minimization to sample the whole space, which was followed by independent simplex and Powell local optimizations and picking the result with a smaller error. The final value of χ^2^ was 6.9.

The previously reported *D*_as_ dispersion function,^11^*D*_as_^10^, is supplemented with parameters for six more elements (B, Al, Si, P, Br, I). Moreover, additional parameters were added to distinguish the carbon sp/sp^2^/sp^3^ hybridization. Just like in the original approach,^11^ unique parameters were assigned to the hydrogen atoms connected to different elements (yielding 13 types of hydrogen atoms in total). This resulted in 30 sets of completely new *C*_*x*_^6^, *C*_*x*_^8^, and β_*x*_ parameters (reported in the Supporting Information, Table S1). The new *D*_as_ dispersion function, *D*_as_^20^, covers most elements of the first three rows (excluding selected metals, see below) plus bromine and iodine.

Four metals from the first three periods (Li, Be, Na, Mg) are not included in the current parametrization (*D*_as_^20^). We have initially tried to include them, but we decided against it for two reasons. First, the errors of *D*_as_^20^ for the dimers containing these metals were very large (see Table S3 of the Supporting Information), in some cases amounting to a couple of kcal·mol^–1^. Note that the results in Table S3 are for the 164 dimers from the final training set plus the additional dimers as listed in Table S3. The calculations of the latter dimers were performed for a set of varying intermolecular distances same as for the former ones. The mean unsigned error (MUE) and the mean unsigned relative error (MURE) for the equilibrium configurations from Table S3 were 1.1 kcal·mol^–1^ and 41.5%, respectively, which should be compared with the analogous errors on the 164 equilibrium configurations from the final training set amounting to 0.1 kcal·mol^–1^ and 5.8%. The second reason for not including metals in our parametrization was that if the dimers containing metals were included, the performance of *D*_as_^20^ on the set of 164 dimers would also slightly deteriorate. The latter problem could have been avoided using specific parameters for all atoms interacting with the four metals, but we have decided just to drop them. These metals are problematic not only for *D*_as_. Table S3 also shows the performance of the D3^50^ function on the same set, in the versions with damping optimized for the HF method and with no damping. The latter approach gives very poor results for most dimers, whereas the former one gives errors about twice as large as *D*_as_^20^ in the variant that includes the four metals.

### 2.3 Training Set of the Dispersion Function

Compared to *D*_as_^10^, the training set used for dispersion benchmarking was extended from 79 to 164 dimers (all of them are listed in Supporting Information, Table S2). Geometries of the 10 dimers containing the bromine and iodine atoms with varying intermolecular distances were taken from the X40 database,^12^ covering noncovalent interactions of molecules containing halogens (10 dimers were chosen out of 18 dimers of this type, see Table S2). Geometries of the remaining 154 dimers were obtained by minimizing interaction energies, free of the basis set superposition error (BSSE), at the second-order of many-body perturbation theory with the Møller–Plesset partitioning of the Hamiltonian (MP2), using the aug-cc-pVTZ basis set^51^ and keeping the MP2/aug-cc-pVTZ optimized geometries of monomers frozen. However, the already optimized 79 dimers from ref (11) were not reoptimized. Ten configurations with varying intermolecular distances *R* between COMs of monomers were generated for each dimer, sampling the whole range between the minimum geometry and the asymptotic region, with the relative orientation of monomers (and monomers’ geometries) kept the same as in the dimer’s equilibrium. An exception was the benzene dimer, where the geometries were taken along the radial cross-section of the surface corresponding to a sandwich configuration rather than to the tilted T-shape minimum^52^ (the latter configuration is not included in our training set). Overall, the training set consisted of 1640 configurations.

The dispersion and exchange-dispersion energies for systems from the X40 dataset (the dimers containing bromine and iodine atoms, see Table S2 of the Supporting Information) were computed using the DFT-SAPT^46−48^ method implemented in MOLPRO^53,54^ (version 2012.1), which employs the PBE0^55,56^ functional and the gradient-regulated asymptotic correction (GRAC).^57^ We have not used the density-fitted version of DFT-SAPT. The aug-cc-pVTZ-PP^58^ basis set, including relativistic pseudopotentials, was chosen for bromine and iodine atoms, and the aug-cc-pVTZ basis was applied for the remaining atoms. The SAPT(DFT) energies for the remaining dimers were obtained using the SAPT2012 program^59^ and the Dalton 2.0^60^ interface. The PBE0 functional^55,56^ with the Fermi–Amaldi–Tozer–Handy (FATH) asymptotic correction^43^ was applied in this case. The aug-cc-pVTZ basis set, supplemented by a 3s3p2d2f set of bond functions with (0.9,0.3,0.1) and (0.6,0.2) exponents for the sp and df functions, respectively, was used. For each monomer, experimental ionization potential (IP) values were taken from ref (61). The reason for applying MOLPRO rather than SAPT codes in the case of the X40 dataset was that the latter codes were used by us with the DALTON 2.0 front-end, which does not include recent relativistic pseudopotentials. All SAPT calculations have been performed in the dimer basis sets.

### 2.4 Test Set for the Dispersion Function

The *D*_as_^20^ function was evaluated on test datasets originating from the following databases:

- the S22^62^ set of 22 hydrogen-bonded, dispersion-bonded, and “mixed” representative biocomplexes,
- the NCCE31/05^63,64^ set of 31 noncovalent complexation energies,
- the NBC10ext^65,66^ set of 10 dispersion-bound bimolecular complexes with off-equilibrium distances (together accounting for 195 dimer geometries),
- the XB51^67^ set of 51 halogen-bonded dimers. Six complexes were excluded from the XB51 database analysis due to the lack of *D*_as_ parameters for lithium (Br_2_–HLi, FI–HLi, CH_3_I–HLi) and palladium (Br_2_–PdHP_2_Cl, FI–PdHP_2_Cl, CH_3_I–PdHP_2_Cl). As a result, only 45 dimers from the XB51 set were included in the evaluation.

The *E*_dispx_^(2)^ energies for these compounds were calculated using the computational protocol described in Section 2.3, using SAPT2012 in the case of S22 and NCCE31/05 datasets, and MOLPRO in the case of the NBC10ext and XB51 datasets. The corresponding IP values for the monomers were obtained from ref (61). However, several monomers from the XB51 dataset lacked the experimental IP values in ref (61), namely, OPH_3_, NBS, and NIS. For these molecules, which are present in the following dimers: Br_2_–OPH_3_, FI–OPH_3_, CH_3_I–OPH_3_, NCH–NBS, NH_3_–NBS, PCH–NBS, NCH–NIS, NH_3_–NIS, and PCH–NIS, the IP values were calculated using PBE0/aug-cc-pVTZ (including pseudopotentials for the bromine and iodine atoms) in Gaussian (version 2016 B-01)^68^ as the difference between DFT energies of a given molecule and its ion (with identical geometries in both cases).

Whenever possible, the *D*_as_^20^ results were compared with the results from the previously published version^11^ of the *D*_as_^10^ dispersion function and other literature parametric dispersion functions. One such very popular function is D3BJ of Grimme et al.^50^ with the Becke–Johnson^69,70^ damping factor. This function is intended to be added to DFT interaction energies and is parametrized specifically for a given density functional. We have considered the versions optimized for the functionals OLYP^71,72^ and revPBE38^73^ (i.e., the revPBE^74^ functional with a 3/8 fraction of the exact exchange), denoted as D3BJ(OLYP) and D3BJ(revPBE38), respectively. One should point out that the name “damping” is misleading in this case since, as pointed out in ref (75), it not only includes the physical damping of the asymptotic expansion due to charge-overlap effects but also corrects DFT interaction energies for errors unrelated to dispersion interactions. Thus, a more appropriate name may be a “switching factor”. We have included the results for D3BJ(NS), where NS stands for “no switching”. All D3BJ computations were performed with the DFT-D3 package (version 3.2, rev. 0).^76^ One more dispersion function included in comparison is DD3S (damped dispersion based on D3 and SAPT),^77^ which uses the D3 long-range coefficients but adjusts two out of three free parameters in the BJ function to SAPT’s *E*_dispx_^(2)^ on the NCCE31/05^63,64^ set of dimers. The remaining parameter was taken from D3BJ(OLYP). Thus, DD3S is constructed partially in the same spirit as *D*_as_.

### 2.5 MED Model with *D*_as_

The proposed MED model (*E*_EL,MTP_^(10)^ + *D*_as_^20^) can be used for the assessment of relative interaction energy between monomers. In particular, the inhibitory activity of protein ligands can be estimated with a low computational cost scaling as *O*(*A*^2^), where *A* stands for the number of atoms.

Here, the *D*_as_^20^ dispersion approximation is applied in the MED model for the examination of the uPA and its five inhibitors, previously described in ref (28). The structures of the uPA complexes, together with MP2 results, were provided by Grzywa et al.,^28^ while the multipole electrostatic contribution was calculated following the description given in Section 2.1. In the case of several protein–inhibitor complexes ranked previously with the MED model encompassing *D*_as_ parameters other than the most recent ones proposed herein, the *D*_as_ contribution was recalculated with the latest *D*_as_^20^ revision. The coordinates of receptor–ligand models and experimental inhibitory activity of the respective complexes were used as described in articles on FAAH,^3^ menin–MLL,^5,13^*Tb*PTR1,^6^ and EphA2–ephA1^4^ complexes. The uPA–inhibitor binding was also analyzed with 10 empirical scoring functions, including AutoDock4^78^ (referred to as AutoDock), AutoDock Vina^79^ (referred to as Vina in what follows), DSX,^80^ RankScore,^81^ PLANTS_PLP_ and PLANTS_CHEMPLP_ (available in PLANTS program^82^), GoldScore, ChemScore, ChemPLP, and ASP (available in GOLD program,^83^ 2020.0 CSD Release). In all of these calculations, the structures of uPA complexes were used in the rescoring mode, i.e., with no docking/optimization. Unless stated otherwise, the default options for all of the rescoring runs were applied. In particular, the PLANTS calculation involved binding site definition encompassing the sphere of a 15 Å radius and the origin associated with the ligand center of mass averaged over all ligands. The latter was also used as the center of a 40 × 40 × 40 point grid in AutoDock calculations. In the case of GOLD rescoring, the cavity was calculated based on the ligand coordinates and the default 6 Å radius (tests with a 15 Å radius yielded identical results concerning the binding score).

In addition, the revised MED model was used on seven hydrogen-bonded (HB) alcohol dimers, previously studied by Hoja et al.:^2^ water (H_2_O)_2_, methanol (MeOH)_2_, ethanol (EtOH)_2_, *n*-propanol (*n*PrOH)_2_, isopropanol (iPrOH)_2_, *n*-butanol (*n*BuOH)_2_, and *tert*-butanol (*t*BuOH)_2_. The coordinates and the corresponding SAPT(DFT) interaction energy values, used as the reference, were taken from ref (2). The multipole electrostatic term was calculated as stated in Section 2.1. However, as in the case of the SAPT calculations performed by Hoja et al.,^2^ the aug-cc-pVTZ basis set^51^ was selected to obtain CAMM. Additional supermolecular interaction energy calculations at the MP2 level of theory were performed using the Gaussian program^68^ (version 2016 B.01) with the same basis set and the counterpoise correction^84^ applied to remove the basis set superposition error (BSSE). We have correlated the *E*_EL,MTP_^(10)^, *D*_as_^20^, and MED data with the experimentally measured affinities of inhibitors. For these “protein” systems, MP2 served as a reference energy for MED. In the case of the HB dimers, no experimental values were available; therefore, *E*_EL,MTP_^(10)^, *D*_as_^20^, and MED were correlated with the SAPT(DFT) energy. In this case, MP2 results are given for comparison only.

The achieved performance was analyzed by means of the coefficient of determination, *R*^2^, calculated for the interaction energy at a given level of theory with respect to the experimentally determined inhibitory activity reported by Grzywa et al.^28^ or with respect to the reported^2^ SAPT(DFT) interaction energies in the case of the HB alcohol dimers
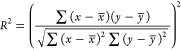
6where *x*(*x̅*) is the (mean) inhibitory activity or the SAPT interaction energy in the case of the HB alcohol dimers and *y*(*y̅*) is the (mean) MED interaction energy. In principle, the interaction energy can be linearly related to the experimentally determined inhibitory potency values (expressed as *p*IC_50_) as long as the inhibitory potency measurements are performed under consistent experimental conditions.^3^

We also computed a statistical predictor, *N*_pred_, which indicates the success rate of a prediction of relative affinities, and is calculated for all pairs of inhibitors as the percentage of concordant pairs with a relative stability of the same sign as in the experimentally determined reference binding potency.^8^ For instance, a concordant pair is a pair of inhibitors *I*_1_, *I*_2_, for which MED(*I*_1_) < MED(*I*_2_) and *p*IC_50_(*I*_1_) > *p*IC_50_(*I*_2_). For the total number of pairs *N*_tot_ and the number of concordant pairs *N*_con_, *N*_pred_ is calculated as
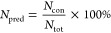
7

Calculations of DISS involved an application of the MED model to structures of enzyme–intermediate and enzyme–substrate complexes of *Comamonas testosteroni* ketosteroid isomerase as derived from the QM/MM simulation reported in ref (85). The DISS value, constituting the difference between the enzyme and reaction intermediate (IS) or substrate (RS) binding energies (DISS = MED(IS) – MED(RS); see ref (23) for further explanation), was evaluated for all KSI amino acid residues in the vicinity of 5 Å of reactants, yielding a total of 22 residues. The total DISS energy was obtained as the sum of residue-wise DISS contributions determined for separate enzyme residue-reaction intermediate/substrate pairs. The dangling bonds resulting from cutting an amino acid residue out of the protein structure were saturated with hydrogen atoms. The multipole electrostatic component of the MED model was calculated with the CAMM expansion, according to the settings given in Section 2.1.

Subsequently, the selected 22 KSI amino acid residues were subjected to the multidimensional scanning procedure, for which the MED model was implemented. Assuming that the amino acid side-chain conformations (rotamers) form a preorganized active site environment,^27^ scanning of the possible rotamers would yield the most optimal rotamer positions, contributing to the lowest possible DISS energy.

The multidimensional scanning protocol involved: (i) loading amino acid rotamers with precomputed CAMM^35^ into specified positions, (ii) excluding rotamers with close contacts (less than 1.7 Å) to the protein backbone, (iii) calculating the MED energy for the retained rotamer-reaction intermediate/substrate pairs, (iv) selecting the most stable positions yielding the lowest DISS energy. To save computational time, possible rotamers were scanned in the presence of the intermediate/substrate and the protein backbone only. However, this approach might lead to a situation where some more catalytically active rotamers clash with nearby residues. In such circumstances, the rotamer scan can be performed with several rotamers simultaneously, yielding the conformations of all scanned residues optimal with respect to both the lowering of the DISS value and the mutual inter-residue interactions. This was the case of the Phe86 residue, for which it was necessary to conduct a simultaneous scan of three rotamers of Leu61, Phe86, and Thr93 residues. Moreover, the currently available multidimensional scanning approach is not applicable to proline or alanine residues; therefore, Pro39, Pro97, and Ala114 residues (present in the initial calculation of total DISS given by KSI active site residues) were not included in this analysis.

## 3 Results and Discussion

### 3.1 *D*_as_ Performance on Training Dataset

The MUE and MURE values of the new *D*_as_^20^ parametrization with respect to the SAPT(DFT) *E*_dispx_^(2)^ values for the training set are equal to 0.1 kcal·mol^–1^ and 5.1%. The performance of *D*_as_^20^ for all configurations of all dimers is shown in Figure S1 in the Supporting Information. As expected from the MURE of 5.1%, typical relative errors are a few percent, and positive and negative errors are evenly distributed. There is one dimer with remarkably small, below 1%, errors across the range of *R*: HBr-CH_3_OH (panel B), but generally, the errors vary quite a lot over the range of *R*, in many cases crossing zero at a small *R* and sometimes also a second time at a large *R*. While the variation with *R* may appear fairly significant for some dimers, these are still within a few percent range for most dimers. There are a couple of outliers with errors at some *R* in excess of 20%: C_2_H_6_–C_2_H_6_ (panel K), PCl_3_–PCl_3_ (AF), and PH_3_–PH_3_ (AG). The fit is particularly bad for PCl_3_–PCl_3_, with all errors above 10% in magnitude. The relative errors are often large in magnitude at large *R*, but the absolute errors always go to zero in this region.

One may observe that distances between some points in the X40x10 set (Figure S1, panels A and B) are very close, only of the order of 0.1 Å. This is due to the fact that for some systems, the authors of ref (12) scaled the distance between two closest atoms, one from monomer A and one from monomer B. This distance is of the order of 2 Å, which (with the step of 0.05 in the scaling factor) leads to the observed high density of points.

### 3.2 Evaluation of *D*_as_ on Test Datasets

The *D*_as_^20^ dispersion function at equilibrium distances was calculated for the complexes found in the S22, NCCE31/05, and XB51 (selected compounds, see Section 2.4) datasets. Additionally, *D*_as_^20^ was calculated for the NBC10ext dataset covering a range of off-equilibrium intermolecular separations (summing up to the 195 dimer geometries included in the analysis). The reference SAPT(DFT) *E*_disp_^(2)^ and *E*_exch-disp_^(2)^ energies were calculated to obtain MUEs and MUREs, which are listed in Table 1 (all energy values are given in Tables S4–S7 of the Supporting Information). Wherever possible, the MUEs and MUREs for the older *D*_as_ version (*D*_as_^10^), ref (11), as well as for the DD3S dispersion term,^77^ are provided. The average errors calculated for selected D3 dispersion functions^50,69^ are also given.

**Table 1:** MUE and MURE Values^a^ for *D*_as_ and Other Approximate Dispersion Energies Relative to *E*_dispx_^(2)^

	benchmark							
	S22		NCCE31/05		NBC10ext		XB51^b^	
method	MUE	MURE	MUE	MURE	MUE	MURE	MUE	MURE
*D*_as_^20^^c^	0.42	5.90	0.18	8.12	0.14	4.06	0.40	10.28
*D*_as_^10^^d^	0.55	7.22	0.14	5.54	0.31	6.01		
DD3S^e^	1.32^f^	24.40^f^	0.57^f^	16.89^f^	1.18	24.98	0.70	17.50
D3BJ(HF)^g^	0.97	13.96	0.77	22.69	0.28	5.31	0.88	18.79
D3BJ(OLYP)^g^	1.76	28.73	0.84	22.43	1.28	30.05	1.01	20.01
D3BJ(revPBE38)^g^	2.29	34.51	1.66	48.25	0.91	15.34	2.42	51.94
D3(NS)^h^	2.78	34.36	1.33	33.67	1.07	20.33	5.52	89.13

a Given in kcal·mol^–1^ and percent, respectively.

b Selected dimers; see Section 2.4 for details.

c Current version of *D*_as_.

d Previously published *D*_as_ version, ref (11).

e Method developed in ref (77).

f Reported or calculated from the data published in ref (77).

g DFT-D3^50^ dispersion term calculated with the BJ damping for the HF level of theory or the listed functional.

h DFT-D3^50^ dispersion term calculated without switching.

The sets S22 and NCCE31/05 partially overlap with the training sets for *D*_as_^10^ and *D*_as_^20^: for both functions, 10 out of 22 S22 dimers and 19 out of 31 NCCE31/05 dimers are in this category (see Table S2, where dimers present in both groups are indicated). Thus, S22 and NCCE31/05 cannot be treated as entirely independent validation sets. Therefore, the errors calculated for these datasets without the overlapping dimers are also given in Tables S4 and S5 of the Supporting Information, along the errors computed for the whole S22 and NCCE31/05 datasets, which are presented in Table 1.

Table 1 shows that for S22, *D*_as_^20^ provides a modest improvement over *D*_as_^10^ in terms of MURE, whereas for NCCE31/05, it gives a 47% larger MURE. This is an anticipated outcome since there are no reasons to expect *D*_as_^20^ to work significantly better than *D*_as_^10^ on systems to which *D*_as_^10^ can be applied (except for the different coefficients for different types of carbon atoms) and since *D*_as_^20^ was fitted to a larger training set, it may be less accurate for the *D*_as_^10^ training dimers included in S22 and NCCE31/05. The main advantage of *D*_as_^20^ is that it can be applied to systems beyond the scope of *D*_as_^10^, as shown in the example of the XB51 set.

For S22 and NCCE31/05, the *D*_as_^10^ and *D*_as_^20^ functions are in a separate class compared to the D3-type functions included in Table 1. In particular, *D*_as_^20^ yields about two times smaller MUREs than these functions. Somewhat surprisingly, DD3S is not the best of such functions for S22, as its MURE is almost two times larger than that of D3BJ(HF). DD3S is 34% better than D3BJ(HF) for NCCE31/05, not surprisingly, since it was optimized on this dataset. The DFT-optimized variants, D3BJ(OLYP) and D3BJ(revPBE38), perform still worse, in particular the latter function. D3(NS) gives smaller errors on S22 and NCCE31/05 than D3BJ(revPBE38) but performs the worst of all methods on XB51.

For the NBC10ext dataset, the *D*_as_^20^ function provides the lowest errors, with *D*_as_^10^ and D3BJ(HF) yielding twice as large MUE and comparable MURE values. The errors associated with the other D3-type functions used in our analysis are roughly an order of magnitude larger than those of *D*_as_^20^. As NBC10ext includes dimers with a broad range of intermolecular separations, good performance of *D*_as_^20^ on this benchmark shows that this function is able to describe dispersion interactions for separations other than the equilibrium one.

The accuracy of *D*_as_^20^ achieved for the selected XB51 dimers is also reasonable, although the MURE value is somewhat larger than for the three other databases. Still, all of the tested D3 variants were outperformed significantly, from a factor of two to almost an order of magnitude in the case of D3(NS).

Similar conclusions concerning the performance of *D*_as_^10^ were reached in ref (86) on the so-called UD-ARL^87^ benchmark set extended by the Ar_2_ and Ar-HF dimers. Table 2 includes comparisons of *D*_as_^20^ with *D*_as_^10^ on several other benchmark sets, namely, S66,^88^ S66x8,^88^ IonHB,^89^ UD-ARL, and S12L^90^ (with the dimer C7a omitted). All values presented in Table 2 are taken from ref (91) (see ref (91) for computational details) and show that *D*_as_^20^ gives systematically more accurate dispersion energies than *D*_as_^10^, although the improvements are generally not large. The only exception is IonHB, where MURE is 0.7% larger in the case of *D*_as_^20^, but MUE is 0.02 kcal·mol^–1^ smaller. Also, the performance on UD-ARL is about the same for both functions. For S66 and S66x8, MUE is decreased by about 0.1 kcal·mol^–1^ and MURE is decreased by about 2%. The improvement is more substantial for S12L, where MUE and MURE went down by 3.5 kcal·mol^–1^ and 7.4%, respectively. MUE of *D*_as_^20^ on S12L may appear fairly large, but one should realize that dispersion energies for these systems, including up to 156 atoms, are tens of kcal·mol^–1^ in magnitude. Small improvements in atomic pairwise contributions to the total dispersion energy may add up to significant differences between the *D*_as_^20^ and *D*_as_^10^ functions for such large complexes. Similar to the NBC10ext dataset, the performance of *D*_as_^20^ on the S66x8, IonHB, and UD-ARL sets with varying separations confirms that *D*_as_^20^ could be used for the description of dispersion in the whole range of interactions.

**Table 2:** MUE and MURE Values^a^ Obtained with *D*_as_^20^ and *D*_as_^10^ Relative to *E*_dispx_^(2)^

	method			
	*D*_as_^20^		*D*_as_^10^	
**benchmark**	**MUE**	**MURE**	**MUE**	**MURE**
S66^88^	0.23	4.13	0.35	6.29
S66x8^88^	0.18	4.93	0.27	6.80
IonHB^89^	0.50	13.76	0.52	13.09
UD-ARL^87^	0.21	6.26	0.22	6.88
S12L^90^	4.64	10.28	8.16	17.64

a Taken from ref (91) and given in kcal·mol^–1^ and percent, respectively.

Reference (91) performed comparisons also for the S22 and NCCE31/05 datasets, and the MUE and MURE values reported there for these benchmarks are somewhat different from the corresponding values given in Table 1: the MUEs differ by 0.05 kcal·mol^–1^ or less, while the MUREs by less than 2.8%. The observed discrepancies are associated with a difference in the *E*_dispx_^(2)^ reference energies occurring mainly due to the fact that calculations of ref (91) used the GRAC asymptotic correction, while refs (10) and (11), where our benchmark values were taken from, used FATH.

### 3.3 Performance of the MED Approach in Modeling Protein–Ligand Interactions

To facilitate the comparison of MED performance across the receptor–ligand systems studied with previous *D*_as_ versions, the MED contribution was recalculated using the *D*_as_^20^ parameters proposed in this work, yielding essentially the same conclusions on the MED scoring abilities. The updated *D*_as_^20^ and MED results concerning FAAH, menin–MLL (I), *Tb*PTR1, EphA2–ephA1, and menin–MLL (II) complexes (Table 3) will be discussed below.

**Table 3:** *E*_EL,MTP_^(10)^, *D*_as_^20^, and MED Performance for Selected Inhibitors in Comparison with the MP2 Results^d^

	*E*_EL,MTP_^(10)^		*D*_as_		MED		MP2	
system	*R*^2^	*N*_pred_	*R*^2^	*N*_pred_	*R*^2^	*N*_pred_	*R*^2^	*N*_pred_
FAAH (ref (3))^a^	0.24	62.8	0.38	74.0	0.45	75.3	0.69	83.1
menin–MLL (I) (ref (13))^a^	0.40	69.3	0.51	77.8	0.78	79.1	0.30	69.9
*Tb*PTR1 (ref (6))^a^	0.23	66.7	0.85	86.7	0.93	86.7	0.79	86.7
EphA2–ephA1 (ref (4))^a^	0.50	77.8	0.44	74.1	0.63	79.6	0.61	77.8
menin–MLL (II) (ref (5))^a^	0.46	74.6	0.12	58.2	0.36	70.9	0.61	81.8
PDE5 (ref (14))	0.20	70.0	0.96	100.0	0.86	90.0	0.90	100.0
uPA (this work)^b^	0.83	80.0	0.97	90.0	0.90	90.0	0.62	80.0
HB dimers (this work)^c^	0.40	52.4	0.96	100.0	0.86	76.2	0.90	100.0

a *E*_EL,MTP_^(10)^ and MP2 results are taken from the original works referenced here, while *D*_as_ and MED values are recalculated following the development of *D*_as_^20^ parameters.

b Inhibitors reported in ref (28).

c Hydrogen-bonded alcohol dimers reported in ref (2).

d The coefficient of determination, *R*^2^, and percentage of successful predictions, *N*_pred_, were calculated with respect to experimentally determined inhibitory potency values or, in the case of the HB dimers, with respect to the SAPT interaction energy values.

As a further validation of the revised *E*_EL,MTP_^(10)^ + *D*_as_ model, this approximate interaction energy measure was computed for selected uPA inhibitors and HB dimers (see Tables S8 and S9 for the *E*_EL,MTP_^(10)^ + *D*_as_ energy values). One should stress that the MED values reported in Tables S8 and S9 can be substantially different from the corresponding SAPT or MP2 interaction energy results, as the *E*_EL,MTP_^(10)^ + *D*_as_ model does not account for other interaction energy terms, e.g., the exchange contribution. Assuming that the neglected terms are approximately constant across the selected systems, the MED results can still be applied to inhibitory activity predictions, being an essential part of drug design protocol. The data gathered in Tables S8 and S9 were used to rank the relative energies (*N*_pred_) or to determine their correlation with experimental values, *R*^2^. The *R*^2^ and *N*_pred_ values obtained for all systems mentioned above are given in Table 3.

In general, the MED model provides reliable results and allows for a rapid estimate of relative binding potency. In the case of EphA2–ephA1, HB dimers, and PDE5, both MED and MP2 models provide comparable results, whereas for the *Tb*PTR1 and uPA systems, MP2 is clearly outperformed by the *E*_EL,MTP_^(10)^ + *D*_as_ approach. Furthermore, for *Tb*PTR1, PDE5, and the HB dimers, the significant correlation of the MED model with the experimental results (or SAPT results in the case of HB dimers) stems essentially from the *D*_as_ dispersion approximation (e.g., for PDE5 *E*_EL,MTP_^(10)^ yields *R*^2^ = 0.20 and *N*_pred_ = 70.0%, whereas *D*_as_^20^ results in *R*^2^ and *N*_pred_ values of 0.96 and 100.0%, respectively; see Table 3). Interestingly, in these cases, MED performs worse than *D*_as_ alone, which is clearly accidental.

The lowest MED coefficient of determination was obtained for the menin–MLL inhibitors with the modified thienopyrimidine scaffold (menin–MLL (II); *R*^2^ = 0.36, see Table 3). It was argued^5^ that the binding of inhibitors for this system was governed by both delocalization and dispersion contributions; therefore, the simple *E*_EL,MTP_^(10)^ + *D*_as_ model cannot achieve satisfactory performance. Neither of the separate MED contributions, *E*_EL,MTP_^(10)^ or *D*_as_, yielded high *R*^2^ values in this case (*R*^2^ = 0.46 and 0.12 for the *E*_EL,MTP_^(10)^ and *D*_as_ terms, respectively).

On the other hand, the MED results obtained for thienopyrimidine ligands (menin–MLL (I); *R*^2^ = 0.78 and *N*_pred_ = 79.1%) appear to be reliable, especially when compared with an unsatisfactory performance of the MP2 level of theory (*R*^2^ = 0.30). This could possibly be due to the presence of shortened protein–inhibitor contacts, as already observed in the case of the uPA system.^28^ It has been shown that the use of force fields in ligand–receptor docking or modeling can lead to artificial shortening of intermolecular contacts, even by 0.5 Å.^9,28^ In such circumstances, the analysis of the interaction energy components at any higher level of theory [e.g., the coupled-cluster method with single, double, and noniterative triple excitations, CCSD(T), or MP2] leads to a dramatic decrease in the quality of relative stability predictions *N*_pred_, resulting from the dominant share of exchange repulsion at distances shorter than the equilibrium geometry obtained from *ab initio* calculations. This problem does not affect MED; therefore, it yields much better *N*_pred_ predictions^3,8,9^ in this case. In contrast to its separate terms, the value of the coefficient of determination obtained with MED is reasonably high for the menin–MLL (I) system, which could be associated with some error cancellation upon the addition of the *E*_EL,MTP_^(10)^ and *D*_as_ terms. Likewise, the MED *N*_pred_ value is the highest for this system (see Table 3).

For the FAAH system, both MP2 and MED results are moderately significant (*R*^2^ = 0.69 and 0.45, respectively), and, similarly to menin–MLL (I), the *R*^2^ of MED attains a higher value than for either of the *E*_EL,MTP_^(10)^ or *D*_as_ contributions. Despite the relatively low *R*^2^ value of the MED model, the corresponding *N*_pred_ factor indicates the correct inhibitor ranking obtained for as many as 75.3% of all of the possible pairs of FAAH–inhibitor complexes.

Overall, the MED method, scaling as *O*(*A*^2^), is dramatically faster than MP2 (which scales as *O*(*N*^5^)) and achieves better scoring for 3 out of 8 systems (menin–MLL (I), *Tb*PTR1, uPA), about the same for another 3 (EphA2–ephA1, PDE5, HB dimers), and worse for only two systems (FAAH, menin–MLL (II)). Thus, the MED performance/cost ratio is excellent.

Noticeably, the computational cost of the nonempirical MED model is as affordable as that of scoring functions used commonly throughout the drug design process.^3^ To further assess the ranking capabilities of the nonempirical MED model, we carried out an analysis of uPA–inhibitor binding with 10 empirical scoring functions, including AutoDock,^78^ Vina,^79^ DSX,^80^ RankScore,^81^ PLANTS_PLP_, PLANTS_CHEMPLP_,^82^ GoldScore, ChemScore, ChemPLP, and ASP.^83^ The correlation coefficients *R* and the *N*_pred_ values along with the numerical data obtained for each particular score are provided in Table S10 in the Supporting Information.

The reason for using *R* to evaluate the correlation here rather than *R*^2^ is that it contains more information due to its sign indicating the direction of a linear relationship. Analysis of the performance of particular models considered herein should account not only for the strength of the relationship but also for its direction. The more potent compounds should be associated with the higher absolute value of the interaction energy, which results in the negative values of the correlation coefficient *R* (e.g., *R* = −0.95 for MED-ranked uPA inhibitors). Unexpectedly, all of the correlation coefficient values obtained with empirical scoring functions tested herein are positive (indicating anticorrelation), ranging from 0.01 to 0.86 for GoldScore and PLANTS_PLP_, respectively (Table S10). The corresponding *N*_pred_ predictivity values do not exceed 60% (compared to 90% for nonempirical MED scoring), with 5 scoring functions featuring *N*_pred_ equal to 50% and the remaining 4 functions yielding an even lower percentage of successful predictions. Empirical scoring functions evaluated herein seem to be incapable of providing reasonable agreement with experimental uPA inhibitory activity, as the interactions with less potent inhibitors are severely overestimated, probably due to the nonphysical shortening of intermolecular distances occurring in uPA–inhibitor complexes.^28^ In contrast, MED results are less sensitive to such structural defects. Remarkably, inaccuracies in receptor–ligand structures are relatively common, resulting from the approximate docking and/or optimization procedures, which additionally calls for scoring methods insensitive to suboptimal intermonomer separation.^8,9,28^ Overall, these results further reinforce the conclusion about favorable predictive abilities of the MED model in comparison to empirical scoring approaches. It should be stated clearly that one limitation of the MED function is the requirement of similar solvation and entropic contributions to binding free energy across the series of ligands considered in a particular analysis. As the MED model accounts only for the enthalpic term of binding free energy, its pertinence requires consistency of the remaining binding free-energy contributions.^4,5,14^

### 3.4 Studying Enzymatic Activity with the MED Model

**Figure 1:**
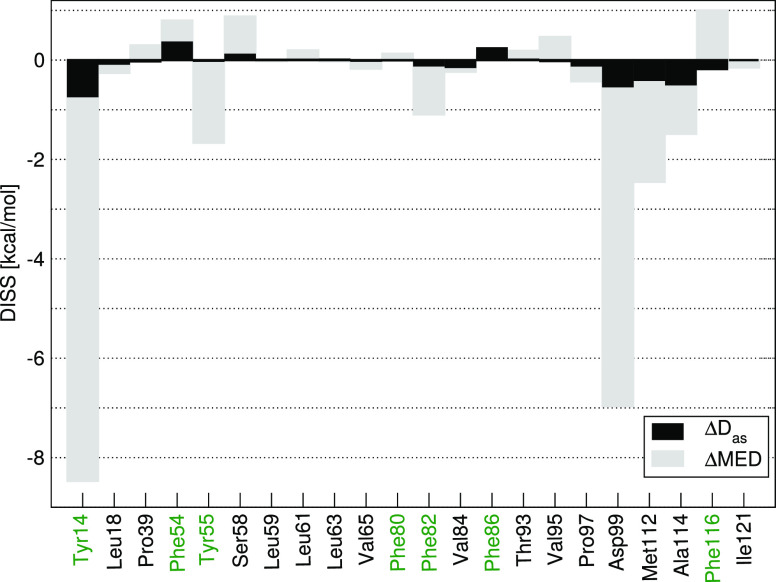
*D*_as_ and MED contribution to the DISS value of particular KSI residues. The residues with an aromatic side chain are shown in green.

The MED model could also be applied for the study of catalytic activity of the enzyme-active site residues, with the ultimate goal of aiding a rational biocatalyst design. Herein, we evaluated the contribution of KSI active site residues to the differential intermediate state stabilization, DISS, expressing the binding of the intermediate by a particular residue relative to the corresponding residue–substrate binding. The KSI-catalyzed reaction consists of isomerization of 5-androstene-3,7-dione to 4-androstene-3,7-dione occurring via two proton transfer steps and the involvement of the dienolate intermediate.^85^ The total DISS value arising from the presence of 22 KSI residues was calculated as the sum of pairwise enzyme residue–substrate and enzyme residue–intermediate interactions. The dispersion part of the total DISS energy obtained with the MED model amounts to −2.1 kcal·mol^–1^, while the total MED DISS energy is −19.4 kcal·mol^–1^ (see Table S11 in the Supporting Information for the DISS values of separate residues). The DISS contributions of 22 KSI residues are shown in Figure 1 for both the MED and *D*_as_ models. It can be seen that most of the KSI active site residues appear to favor the catalysis by preferential binding of the reaction intermediate.

The largest contribution to DISS results from the presence of the Tyr14 and Asp99 residues, in agreement with the site-directed mutagenesis data,^92,93^ demonstrating a lower catalytic activity of KSI mutants with substitutions that involved these residues. Other residues contributing favorably to the KSI-catalyzed reaction include Met112, Tyr55, Ala114, and Phe82. A minor adverse impact on catalysis originates from, e.g., the Phe116, Ser58, and Phe54 residues, exhibiting positive values of the DISS energy (Figure 1), indicating a destabilization of the reaction intermediate compared to the substrate.

Most of the total dispersion contribution to the DISS energy arises from the residues with the highest, favorable impact on the activation barrier lowering (i.e., Tyr14, Asp99, Ala114, and Met112). The aromaticity of a residue (see Figure 1 for residue labels marked in green) does not necessarily imply a larger dispersive contribution of a given residue, as Asp99 or Ala114 residues, bearing no aromatic side chain, are among the ones characterized by the largest absolute Δ*D*_as_ values. Except for a relatively insignificant differential intermediate destabilization resulting from dispersive interactions between the reaction intermediate or substrate and, e.g., the Phe54 or Phe86 residues (see Figure 1), the dispersive interactions generally support the catalytic influence of the enzyme-active site by differential binding of the intermediate state.

The side-chain conformations of amino acid residues are likely related to the enzyme catalytic affinity.^94^ In particular, the influence of some designed mutations on the enzyme activity could only be explained with a detailed insight into dynamical preorganization of the active site acquired from extensive molecular dynamics simulations.^95^ To aid the biocatalyst design, there is a need for a conformation screening methodology that would yield side-chain rotamers optimal with respect to the catalytic activity and would be affordable enough to be applied in the high-throughput scanning of possible side-chain conformations. The feasibility of the latter has already been proven with the use of CAMM library^35^ of amino acid side-chain rotamers.^36^ Herein, we demonstrate that the MED model in conjunction with precalculated CAMM values of side-chain rotamers^35,36^ is capable of the rapid determination of catalysis-aiding rotamers.

**Figure 2:**
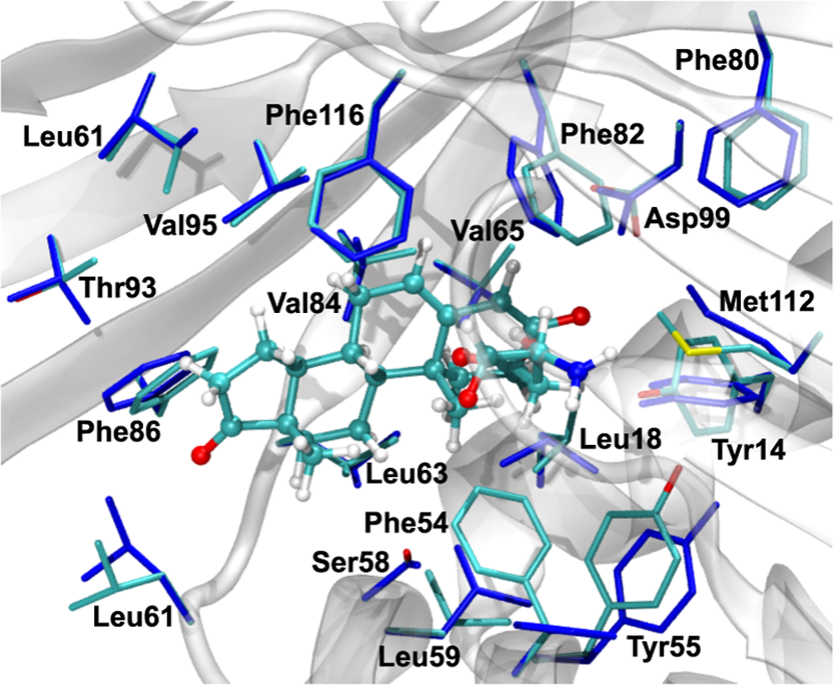
Rotamers of KSI active site residues optimized for differential intermediate state stabilization (in blue) in comparison with side-chain conformations present in the original structure. The reaction intermediate is shown in ball-and-stick representation.

The KSI active site residues included in Figure 1 were subjected to the multidimensional search of rotamers, according to the protocol described in Section 2.5. The side-chain rotamers yielding the lowest possible DISS value (see Table S12 for details), as obtained from the MED model, are presented in Figure 2.

In general, the DISS values arising from the presence of KSI residues with optimized rotamers increased in magnitude, with the total DISS of −20.8 kcal·mol^–1^, lower by 1.4 kcal·mol^–1^ compared to residue conformations present in the original KSI structure (see Table S12). The observed lowering of the DISS value resulting from the rotamer scan does not seem to be significant, but it should be emphasized that the current analysis was performed for a highly efficient, naturally evolved enzyme, whereas in the case of the enzyme (re)design, the ability to determine the side-chain rotamer yielding the greatest catalytic effect starting from a suboptimal conformation of a mutated residue might prove crucial.^27^ As demonstrated for the KSI-catalyzed reaction, including the *D*_as_ dispersion in the MED model further extends the applicability of the differential transition state stabilization and a multidimensional scan of the side-chain conformational space to enzymes with a non-negligible contribution of dispersive interactions.

## 4 Summary

We have developed an extension of the previously published *D*_as_^10^ (ref (11)) dispersion function, *D*_as_^20^. The current reparametrization, yielding a set of completely new *D*_as_^20^ parameters, allows computing an approximate dispersion energy for systems with six additional elements: B, Al, Si, P, Br, I. It also distinguishes the carbon sp/sp^2^/sp^3^ hybridizations and, similarly to the *D*_as_^10^ version, differentiates the hydrogen atom types based on their connection to a given element.

An important outcome of this new development is that halogen-bonded systems of biological significance can be studied with the revised *D*_as_^20^ function. As indicated by the relatively low MUE and MURE values relative to the *ab initioE*_dispx_^(2)^ energies obtained with the new *D*_as_^20^ expression in the case of the S22, NCCE31/05, NBC10ext, XB51, S66, S66x8, IonHB, UD-ARL, and S12L benchmarks, the accuracy of this approach is rather satisfactory, in particular in comparison to other dispersion functions of similar form. *D*_as_^20^ can also be used in biomolecular force fields, replacing the current *C*_6_^*ab*^/*r*_*ab*_^6^ terms and, of course, readjusting some other parameters. This is of importance due to the recent finding of shortcomings of this term in such force fields.^96−99^ In particular, the account of the dispersion interactions has recently been found critical for describing disordered proteins.^100^ Finally, *D*_as_^20^ is applicable in the new generation of biomolecular force fields fitted simultaneously to experiment and SAPT interaction energy components.^101−103^

A deficiency of *D*_as_^20^ is that still only selected atoms are parametrized, whereas methods based on the D3 dispersion provide parameters for every element up to *Z* = 94 (although the D3 results for molecules that include alkali and alkaline earth metals are essentially useless, see Table S3 of the Supporting Information). Nevertheless, for the majority of applications in biochemistry, all needed atoms are covered by the *D*_as_^20^ function. For such systems, *D*_as_^20^ will likely approximate the dispersion interaction better than any published function of this type.

The revised *D*_as_^20^ expression was applied within the MED (*E*_EL,MTP_^(10)^ + *D*_as_) model for the scoring of uPA inhibitors and hydrogen-bonded dimers. The MED scoring is based on the assumption that the relative protein–inhibitor interaction energies near their equilibrium separations can be estimated from the MED interaction energies. In addition, the interaction energy can be linearly related to the ligand affinity. In a series of investigated protein–ligand systems, this approach was able to provide a significant correlation between the experimental potency and the computed *E*_EL,MTP_^(10)^ + *D*_as_ interaction energy. Given that an accurate and (at the same time) efficient description of enzyme–inhibitor systems are not straightforward, these results are of vital importance for the process of design and scoring of novel inhibitors without involving any empirical factors. The significance of the MED model is further reinforced by its favorable performance in ligand ranking compared to a number of commonly used empirical scoring functions. The use of MED is particularly advantageous in the case of geometries suffering from insufficient accuracy, indicating that MED is very robust with respect to structural deficiencies. Featuring the computational cost as low as that of empirical scoring functions, the MED model has been shown to yield the results of the quality similar (or even better) to that of the top-scoring approaches applied commonly in ligand design projects.

The MED model can also be applied to study the enzyme-active site residues to facilitate rational biocatalyst (re)design. For this purpose, we have implemented the MED model into our multidimensional rotamer scan algorithm. With the KSI system as an example, we have shown herein that accounting for dispersion in the process of obtaining the DISS energy through a multidimensional search of amino acid rotamers is possible, therefore extending the applicability of this method to enzymes in which dispersion interactions are non-negligible.

## Supporting Information Available

Details regarding the dispersion fit, the list of *D*_as_^20^ parameter, the results obtained for the benchmark datasets, and the MED results obtained for the biological systems; after this work has been finished, we found that our code incorrectly identifies a C atom bound to *k* Cl, Br, or I atoms as of sp^*n*–*k*^ type rather than sp^*n*^; since this mistake occurred during the optimization, the C^6^, C^8^, and β coefficients for different hybridizations of C have adjusted to mitigate the effects of this mistake and the dispersion function was able to achieve the good performance described earlier despite this mistake; however, for cases with three such halogens bound to C (no such cases appeared in any calculations in the present paper), there are no appropriate coefficients and the code uses the universal (i.e., hybridization independent) carbon coefficient from ref (11) (PDF)
Python script to calculate the *D*_as_^20^ dispersion function (TXT)
