# Supplementary material for: Extension of an Atom–Atom Dispersion Function to Halogen Bonds and Its Use for Rational Design of Drugs and Biocatalysts
Source: J Phys Chem A. 2021 Feb 23;125(8):1787–99. doi: 10.1021/acs.jpca.0c11347 (PMC8028329; doi:10.1021/acs.jpca.0c11347)
Supplement: Supplementary file 1 — jp0c11347_si_001.pdf [file jp0c11347_si_001.pdf]

Supporting Information: Extension of an Atom-Atom Dispersion Function to Halogen Bonds and Its Use for Rational Design of Drugs and Biocatalysts

Wiktoria Jedwabny,[*,†] Edyta Dyguda-Kazimierowicz,[*,†] Katarzyna Pernal,[‡] Krzysztof Szalewicz,[¶] and Konrad Patkowski[§]

†*Department of Chemistry, Wrocław University of Science and Technology, Wybrzeże Wyspiańskiego 27, 50-370 Wrocław, Poland*

‡*Institute of Physics, Łódź University of Technology, Wólczańska 219, 90-924 Łódź, Poland*

¶*Department of Physics and Astronomy, University of Delaware, Newark, Delaware 19716, United States*

§*Department of Chemistry and Biochemistry, Auburn University, Auburn, Alabama 36849, United States*

E-mail: Wiktoria.Jedwabny@pwr.edu.pl,JedwabnyW@gmail.com; Edyta.Dyguda@pwr.edu.pl

1 Details of $E^{(2)}_{\mathrm{dispx}}$ calculations

The majority of the $E^{(2)}_{\mathrm{dispx}}$ values were obtained with SAPT(DFT) method implemented in SAPT2008 program,[S1] which applied PBE0 functional[S2,S3] with the Fermi-Amaldi-Tozer-Handy asymptotic correction,[S4] and the aug-cc-pVTZ basis set,[S5] supplemented by a 3s3p2d2f set of bond functions with (0.9,0.3,0.1) and (0.6,0.2) exponents for sp and df functions, respectively. The energies for systems from the X40x10[S6] (training dataset), NBC10ext[S7] and XB51 Kozuch and Martin[S8] datasets (test datasets) were calculated with DFT-SAPT[S9–S11] method implemented in MOLPRO[S12,S13] (version 2012.1) without density fitting, which applied PBE0 functional and the gradient-regulated asymptotic correction[S14] and the aug-cc-pVTZ basis set or, in the case of bromine and iodine atoms, the aug-cc-pVTZ-PP[S15] basis set (accounting for relativistic pseudopotentials). Experimental ionization potential (IP) values, needed for the asymptotic correction, were obtained from Ref. S16. However, OPH_3, NBS, and NIS monomers from XB51 dataset lacked the experimental IP values in Ref.,[S16] and for these molecules, present in the following dimers: $Br_2 - OPH_3$, $FI - OPH_3$, $CH_3I - OPH_3$, NCH − NBS, $NH_3 - NBS$, PCH − NBS, NCH − NIS, $NH_3 - NIS$, PCH − NIS, IP values were calculated in Gaussian (version 2016 B-01)[S17] using the PBE0/aug-cc-pVTZ (including pseudopotentials for bromine and iodine atoms) method.

2 Details of the dispersion fit

The training set used for the determination of D^{20}_{as} parameters, shown in Table S1, consisted of 164 dimers (see Table S2). Geometries of the training set dimers with bromine and iodine atoms were taken from the X40 database,[S6] and included four geometries with the center-of-mass (COM) separation (R) smaller than at the minimum, and five geometries with the COM separation larger than at the minimum (together yielding ten different configurations for each dimer). Geometries of the remaining dimers were optimized at the second order Moeller-Plesset MP2/aug-cc-pVTZ[S5] level of theory (several minimum geometries were obtained from the S22 or NCCE31/05 datasets, as indicated in Table S2). For these dimers, ten different radial geometries corresponding to the same angular configuration were provided. In particular, the minimum geometry was accompanied by two geometries with the COM separation smaller than at the minimum, and seven geometries with the COM separation larger than at the minimum (up to 10Å). Overall, there were 1640 configurations in the training set, and the MUE and MURE errors associated with the obtained D^{20}_{as} values were equal to 0.1 $\mathrm{kcal \cdot mol^{-1}}$ and 5.1%, respectively.

The D^{20}_{as} parameters are given in Table S1. The C^6_x and C^8_x coefficients are given in the units of $\mathrm{J \cdot nm^6 \cdot mol^{-1}}$ and $\mathrm{J \cdot nm^8 \cdot mol^{-1}}$, respectively. The values of β_x are given in $\mathrm{bohr^{-1}}$. To obtain the values of C^6_x and C^8_x in atomic units, the corresponding values need to be multiplied by 17.34525495 and 6194.102092, respectively.

Table S1: List of D_{as}^{20} dispersion expression parameters.

Element	$C_x^{6\,a}$	$C_x^{8\,b}$	$\beta_x{}^c$
H-H	0.199730	0.010225	1.898977
H-B	0.625058	0.004286	2.022739
H-C	0.216319	0.002841	1.737714
H-N	0.117956	0.012842	1.456906
H-O	0.155250	0.003195	1.623847
H-F	0.028773	0.005962	1.618463
H-Al	0.984668	0.060477	1.828202
H-Si	0.957855	0.011257	1.627960
H-P	0.338996	0.296480	0.589819
H-S	0.405991	0.011234	1.416878
H-Cl	0.095880	0.008433	1.660460
H-Br	0.185489	0.020926	1.386093
H-I	0.185411	0.045112	1.310652
He	0.071774	0.004783	2.225149
B	0.003104	0.264031	1.695239
C_{sp^1}	1.297139	0.171713	1.951721
C_{sp^2}	0.914964	0.268786	1.658960
C_{sp^3}	0.461576	0.235496	1.780635
N	1.427069	0.070811	2.396253
O	0.723590	0.052314	2.368908
F	0.900101	0.015629	2.458106
Ne	0.261125	0.028508	2.132240
Al	0.415583	0.900131	1.133178
Si	0.009843	0.894086	2.726511
P	0.890484	2.415552	1.634346
S	4.793738	0.825826	2.279597
Cl	6.254113	0.691238	1.643112
Ar	3.331510	0.509268	1.843843
Br	9.995193	1.169732	1.572187
I	21.167203	2.745814	1.463387

[a]In units of $J \cdot nm^6 \cdot mol^{-1}$. Multiply by 17.34525495 to obtain C_x^6 in atomic units.
[b]In units of $J \cdot nm^8 \cdot mol^{-1}$. Multiply by 6194.102092 to obtain C_x^8 in atomic units.
[c]In units of $bohr^{-1}$.

Table S2: Monomers used for the fit of D_{as}^{20} parameters to the benchmark $E_{\mathrm{dispx}}^{(2)}$ energies. Dimers denoted in blue, red or green overlap with the S22, NCCE31/05 or both of these test sets, respectively.

A	B
$AlCl_3$	Ar, He, Ne
AlF_3	AlF_3, Ar, He, Ne
AlH_3	AlH_3, Ar, He, Ne
Ar	Ar, C_2H_2, C_2H_6, CH_3OH, CH_4, CO_2
BCl_3	Ar, BCl_3, He, Ne
BF_3	Ar, BF_3, He, Ne
BH_3	Ar, BH_3, He, Ne
C_2H_2	C_2H_2, ClF, CO_2, HCl, He, Ne, NH_3
C_2H_4	Ar, C_2H_4, CH_4, F_2, H_2O, NH_3
C_2H_6	C_2H_2, C_2H_6, CH_3OH, NH_3, Ne
C_6H_6	Ar, C_6H_6[a], CF_3Br, CF_3I, CH_4, H_2O, He, Ne
CH_3Br	C_6H_6, CH_2O
CH_3I	C_6H_6, CH_2O
CH_3OH	CH_3OH, CH_4, H_2O, HBr, HI, Ne, NH_3
CH_4	Br_2, C_2H_2, C_2H_6, CH_4, I_2, Ne, NH_3
CO_2	C_2H_4, C_2H_6, CH_3OH, CH_4, CO_2
H_2	Ar, H_2, H_2O, He, Ne
H_2O	Ar, C_2H_2, C_2H_6, CH_4, ClF, CO_2, H_2O
H_2S	Ar, C_2H_2, C_2H_6, CH_3OH, CO_2, H_2O, HF, H_2S
HCl	Ar, CH_3OH, CH_4, CO_2, H_2O, H_2S, HCl, He
$HCONH_2$	$HCONH_2$
HCOOH	HCOOH
HF	Ar, C_2H_2, CH_3OH, CH_4, CO_2, H_2O, HF, N_2
He	Ar, C_2H_6, CH_3OH, CH_4, CO_2, H_2O, H_2S, HF, He
NH_3	Ar, ClF, H_2S, He, Ne, NH_3, CO_2, H_2O
Ne	Ar, CO_2, H_2O, H_2S, HCl, HF, Ne
PCl_3	Ar, He, Ne, PCl_3
PF_3	Ar, He, Ne, PF_3
PH_3	Ar, He, Ne, PH_3
pyrazine	pyrazine
$SiCl_4$	Ar, He, Ne
SiF_4	Ar, He, Ne, SiF_4
SiH_4	Ar, He, Ne, SiH_4

[a]Benzene dimer in sandwich configuration.

3 Detailed numerical results

Table S3: $E^{(2)}_{\mathrm{dispx}}$, D_{as} and D3 correction energies[a] calculated for excluded metal complexes at equilibrium distances[b].

Dimer	$E^{(2)}_{\mathrm{dispx}}$	D_{as}	D3(NS)[c]	D3BJ(HF)[d]
Ar – BeH_2	-0.8	-0.8	-0.7	-0.7
Ar – BeO	-0.9	-0.5	-0.5	-0.5
Ar – Li_2O	-0.6	-1.0	-6.1	-2.6
Ar – LiH	-0.5	-0.9	-6.0	-2.8
Ar – MgH_2	-1.0	-0.8	-1.2	-1.2
Ar – MgO	-1.6	-1.1	-0.9	-0.8
Ar – Na_2O	-0.4	-0.7	-1.9	-1.4
Ar – NaH	-0.5	-0.3	-2.1	-1.7
Be – Be	-7.6	-7.1	-17.3	-9.3
BeH_2 – BeH_2	-17.3	-16.6	-70.4	-8.7
BeO – BeO	-7.3	-8.6	86.6	-3.9
H_2 – BeH_2	-1.0	-1.0	-0.5	-0.9
H_2 – BeO	-1.2	-0.8	-0.8	-0.7
H_2 – Li_2O	-3.0	-2.3	53.5	-3.4
H_2 – LiH	-0.5	-0.9	56.4	-2.5
H_2 – MgH_2	-1.7	-1.6	9.2	-2.2
H_2 – MgO	-1.9	-1.4	-1.2	-1.0
H_2 – Na_2O	-6.3	-4.1	51.3	-4.0
H_2 – NaH	-0.8	-0.3	-0.2	-0.3
He – BeH_2	-0.1	-0.1	-0.1	-0.1
He – BeO	-0.1	-0.1	-0.1	-0.1
He – Li_2O	-0.2	-0.5	-2.9	-0.9
He – LiH	-0.2	-0.4	-1.5	-0.9
He – MgH_2	-0.1	-0.1	-0.1	-0.1
He – MgO	0.0	0.0	0.0	0.0
He – Na_2O	0.0	0.0	-0.1	0.0
He – NaH	-0.1	-0.1	-0.2	-0.2
Li_2O – Li_2O	-7.3	-6.8	-192.5	-13.4
LiH – LiH	-9.8	-2.3	-92.3	-7.0
Li – Li	-0.8	-0.8	-1.2	-1.1
MgH_2 – MgH_2	-5.2	-2.9	-9.9	-3.8
Mg – Mg	-2.9	-2.7	-4.5	-3.7
MgO – MgO	-34.2	-51.7	7.7	-6.5
Na_2O – Na_2O	-6.2	-6.4	-100.4	-12.8
NaH – NaH	-8.3	-0.6	-47.1	-7.0
Na – Na	-0.5	-0.5	-0.9	-0.9
Ne – BeH_2	-0.2	-0.2	-0.2	-0.2

Table S3: continued from previous page.

Dimer	$E^{(2)}_{\text{dispx}}$	D_{as}	D3(NS)	D3BJ(HF)
Ne – BeO	-0.2	-0.1	-0.1	-0.1
Ne – Li_2O	-0.3	-0.6	-3.0	-1.2
Ne – LiH	-0.3	-0.5	-2.4	-1.2
Ne – MgH_2	-0.2	-0.2	-0.3	-0.3
Ne – MgO	-0.4	-0.3	-0.2	-0.2
Ne – Na_2O	-0.2	-0.3	-0.6	-0.5
Ne – NaH	-0.2	-0.1	-0.5	-0.6
MUE/MURE:		1.1/41.5	18.5/664.9	1.8/107.5

[a]In units of $\text{kcal} \cdot \text{mol}^{-1}$.

[b]Benchmark excluded from final D_{as} parametrization.

[c]D3 correction calculated with no switching.

[d]D3 correction calculated with BJ damping for the HF level of theory.

Table S4: $E^{(2)}_{\text{dispx}}$, D^{20}_{as}, and D3 energies[a] calculated for each dimer from the S22 database[b]. Corresponding MUE and MURE values are given.

Dimer	$E^{(2)}_{\text{dispx}}$	D^{20c}_{as}	D3BJ(HF)[d]	D3BJ(OLYP)[d]	D3BJ(revPBE38)[d]	D3(NS)[e]
2-pyridoxine – 2-aminopyridine	-9.7	-9.7	-7.1	-8.0	-4.2	-14.4
adenine – thymine (stack)	-16.0	-19.1	-15.3	-21.3	-12.9	-9.8
adenine – thymine (wc)	-9.8	-10.1	-7.4	-8.4	-4.4	-14.4
NH_3 dimer	-2.2	-2.2	-1.9	-2.2	-1.0	-2.6
C_6H_6 – NH_3	-2.9	-2.9	-2.9	-3.9	-2.2	-2.9
C_6H_6 dimer (c2h)	-8.0	-7.7	-7.0	-10.0	-6.4	-5.4
C_6H_6 dimer (c2v)	-4.6	-4.5	-4.6	-6.2	-3.7	-4.4
C_6H_6 – HCN	-3.6	-4.0	-4.3	-5.5	-2.9	-4.5
C_6H_6 – CH_4	-2.9	-2.6	-2.7	-3.6	-2.2	-2.7
C_6H_6 – H_2O	-2.8	-2.8	-3.1	-4.0	-2.2	-3.4
C_2H_4 dimer	-2.6	-2.7	-2.9	-3.8	-2.1	-3.1
C_2H_4 – C_2H_2	-1.4	-1.3	-1.4	-1.8	-1.0	-1.4
$HCONH_2$ dimer	-7.8	-7.8	-5.5	-5.9	-2.8	-13.3
HCOOH dimer	-9.5	-9.5	-6.1	-6.2	-2.7	-23.2
indole – C_6H_6 (stack)	-11.7	-11.6	-10.2	-14.4	-9.2	-6.3
indole – C_6H_6 (t-shape)	-6.3	-6.9	-6.8	-8.7	-5.1	-6.7
CH_4 dimer	-1.1	-1.0	-1.0	-1.4	-0.9	-1.0
phenol dimer	-6.5	-6.6	-5.8	-7.3	-4.1	-7.9
pyrazine dimer	-8.4	-8.9	-7.6	-10.8	-6.7	-5.0
uracil dimer (hb)	-9.2	-9.5	-6.4	-7.1	-3.5	-16.9
uracil dimer (stack)	-11.1	-13.3	-10.9	-15.1	-9.1	-10.2
H_2O dimer	-2.4	-2.3	-1.8	-1.8	-0.7	-3.9
MUE/MURE:		0.4/5.9	1.0/14.0	1.8/28.7	2.3/34.5	2.8/34.4
MUE/MURE$_{12}$:[f]		0.6/7.1	1.0/11.8	2.1/28.8	2.5/31.2	1.5/15.2

[a]In units of $\text{kcal} \cdot \text{mol}^{-1}$.

[b]Benchmark reported in Ref. S18.

[c]D_{as} revision reported herein.

[d]D3[S19] dispersion term calculated with BJ damping for the HF level of theory or given functional.

[e]D3[S19] dispersion term calculated without switching.[S20]

[f]Errors calculated for the S22 dataset restricted to dimers not present in the D_{as} training set. See Table S2 for the overlapping dimers.

Table S5: $E^{(2)}_{\rm dispx}$, $D^{20}_{\rm as}$, and D3 energies[a] calculated for each dimer from the NCCE31/05 database[b]. Corresponding MUE and MURE values are given.

Dimer	$E^{(2)}_{\rm dispx}$	$D^{20}_{\rm as}$[c]	D3BJ(HF)[d]	D3BJ(OLYP)[d]	D3BJ(revPBE38)[d]	D3(NS)[e]
C_2H_2 dimer	-1.4	-1.3	-1.3	-1.8	-1.1	-1.3
C_2H_2 – ClF	-5.0	-4.9	-4.1	-4.3	-1.9	-2.1
C_2H_4 dimer	-2.6	-2.7	-2.9	-3.8	-2.1	-3.1
C_2H_4 – F_2	-1.6	-1.3	-0.9	-1.2	-0.6	-0.9
C_6H_6 – Ne	-1.0	-0.9	-0.8	-1.1	-0.7	-0.6
CH_3Cl – HCl	-3.6	-3.5	-3.4	-3.9	-1.9	-4.8
CH_3SH – HCl	-4.6	-4.5	-4.1	-4.7	-2.3	-6.2
CH_4 dimer	-1.3	-1.2	-1.2	-1.6	-1.0	-1.2
CH_4 – Ne	-0.3	-0.3	-0.3	-0.4	-0.2	-0.3
H_2O – ClF	-4.0	-3.8	-2.5	-2.5	-1.1	-3.1
H_2O dimer	-2.3	-2.1	-1.6	-1.7	-0.7	-3.6
H_2S dimer	-2.1	-1.9	-1.7	-2.2	-1.2	-2.0
H_2S – HCl	-3.0	-2.7	-2.5	-2.9	-1.4	-3.7
HCN – CH_3SH	-2.7	-2.8	-1.9	-2.2	-1.1	-2.6
HCN – ClF	-4.2	-4.0	-2.8	-3.4	-1.8	-3.2
$HCONH_2$ dimer	-7.4	-7.4	-2.8	-2.7	-1.2	-2.9
HCOOH dimer	-8.8	-8.7	-5.3	-5.7	-2.7	-12.3
HCl dimer	-2.1	-1.9	-5.8	-6.0	-2.6	-20.0
HF dimer	-1.7	-1.6	-0.1	-0.1	-0.1	-0.1
He – Ar	-0.1	-0.1	-0.1	-0.1	0.0	-0.1
He – Ne	-0.1	-0.1	-1.2	-1.2	-0.4	-3.4
NH_3 – Cl_2	-4.7	-3.9	-0.2	-0.3	-0.2	-0.2
NH_3 – ClF	-9.2	-8.7	-0.1	-0.2	-0.1	-0.1
NH_3 – F_2	-1.8	-1.3	-2.7	-2.9	-1.4	-2.2
NH_3 – H_2O	-3.0	-2.7	-4.8	-4.2	-1.7	-7.4
NH_3 dimer	-2.1	-1.8	-0.7	-0.9	-0.4	-0.8
Ne – Ar	-0.3	-0.2	-2.1	-2.1	-0.9	-4.1
Ne dimer	-0.2	-0.1	-1.5	-1.7	-0.8	-2.1
C_6H_6 dimer (P)	-5.8	-5.5	-5.1	-7.2	-5.0	-3.9
C_6H_6 dimer (S)	-4.7	-4.4	-4.1	-5.8	-4.1	-3.2
C_6H_6 dimer (T)	-4.1	-4.0	-4.1	-5.5	-3.4	-3.8
MUE/MURE:		0.2/8.1	0.8/22.7	0.8/22.4	1.7/48.3	1.3/33.7
MUE/MURE_{12}:[f]		0.2/8.2	0.6/19.9	0.7/21.6	1.5/49.8	1.1/36.4

[a] In units of kcal · mol^{-1}.

[b] Benchmark reported in Ref. S21.

[c] $D_{\rm as}$ revision reported herein.

[d] D3[S19] dispersion term calculated with BJ damping for the HF level of theory or given functional.

[e] D3[S19] dispersion term calculated without switching.[S20]

[f] Errors calculated for the NCCE31/05 dataset restricted to dimers not present in the $D_{\rm as}$ training set. See Table S2 for the overlapping dimers.

Table S6: $E^{(2)}_{\mathrm{dispx}}$, D^{20}_{as}, D3, and DD3S energies[a] calculated for each dimer from the XB51 database[b]. Corresponding MUE and MURE values are given.

Dimer	$E^{(2)}_{\mathrm{dispx}}$	$D^{20\,c}_{\mathrm{as}}$	D3BJ(HF)[d]	D3BJ(OLYP)[d]	D3BJ(revPBE38)[d]	D3(NS)[e]	DD3S[f]
Br_2 – FCCH	-1.1	-1.2	-0.9	-1.2	-0.7	-0.9	-1.2
Br_2 – CH_3F	-3.0	-3.2	-2.5	-2.9	-1.5	-3.0	-3.4
Br_2 – NCH	-3.2	-3.0	-2.6	-2.7	-1.3	-2.0	-3.3
Br_2 – NH_3	-5.5	-5.1	-3.9	-3.8	-1.8	-2.9	-4.7
Br_2 – OCH_2	-4.0	-4.1	-3.5	-3.7	-1.8	-3.0	-4.5
Br_2 – PCH	-2.5	-2.0	-2.7	-2.9	-1.6	-3.3	-3.4
Br_2 – pyridine	-7.6	-7.5	-5.2	-5.6	-2.9	16.5	-6.4
CH_3I – FCCH	-0.5	-0.5	-0.3	-0.4	-0.3	-0.3	-1.3
CH_3I – CH_3	-4.4	-5.2	-3.8	-3.7	-1.8	-5.3	-3.1
CH_3I – NCH	-6.3	-7.0	-4.9	-3.9	-1.8	35.2	-2.5
CH_3I – NH_3	-8.8	-9.7	-6.7	-5.5	-2.4	-10.5	-3.0
CH_3I – OCH_2	-6.3	-7.2	-5.1	-4.5	-2.1	21.8	-4.0
CH_3I – PCH	-4.8	-4.5	-5.7	-4.2	-2.2	-5.7	-3.1
CH_3I – pyridine	-11.9	-13.9	-7.9	-7.3	-3.8	54.2	-4.8
FI – FCCH	-1.1	-1.1	-0.9	-1.3	-0.7	-0.9	-0.4
FI – CH_3F	-2.6	-2.6	-2.3	-2.9	-1.6	-2.2	-4.6
FI – NCH	-2.2	-1.9	-1.9	-2.3	-1.2	-1.3	-5.1
FI – NH_3	-3.0	-2.6	-2.3	-2.8	-1.5	-1.0	-7.4
FI – OCH_2	-3.1	-3.0	-3.0	-3.6	-1.9	-2.1	-5.7
FI – PCH	-2.1	-1.6	-2.4	-2.8	-1.6	-2.8	-5.4
FI – pyridine	-4.4	-4.0	-3.7	-4.6	-2.6	0.7	-8.7
NCH – F_3CI	-2.9	-2.7	-2.6	-2.8	-1.4	-1.2	-3.2
NCH – FBr	-5.5	-5.9	-4.1	-3.4	-1.5	10.2	-4.6
NCH – FCl	-3.7	-4.0	-2.8	-2.7	-1.2	-2.7	-3.5
NCH – PhBr	-1.9	-1.6	-1.5	-1.9	-1.0	-1.4	-2.1
NCH – PhI	-2.4	-2.1	-2.0	-2.4	-1.3	-1.4	-2.7
NH_3 – F_3CI	-4.2	-3.8	-3.3	-3.6	-1.8	-1.4	-4.2
NH_3 – FBr	-8.9	-9.5	-5.9	-4.8	-2.0	-10.4	-6.7
NH_3 – FCl	-7.4	-7.9	-4.6	-4.1	-1.7	-6.0	-5.6
NH_3 – PhBr	-2.6	-2.1	-1.8	-2.3	-1.2	-1.3	-2.4
NH_3 – PhI	-3.3	-2.8	-2.5	-3.0	-1.6	-1.1	-3.2
PCH – F_3CI	-2.2	-1.8	-2.6	-2.9	-1.7	-3.0	-3.2
PCH – FBr	-4.1	-3.7	-4.5	-3.7	-1.8	-5.7	-4.7
PCH – FCl	-2.8	-2.6	-2.9	-3.0	-1.5	-3.8	-3.6
PCH – PhBr	-2.0	-1.4	-2.0	-2.5	-1.4	-2.4	-2.7
PCH – PhI	-2.2	-1.7	-2.5	-2.9	-1.7	-2.9	-3.1
BrBr – OPH_3	-5.1	-5.1	-4.8	-4.8	-2.4	-6.0	-5.9
FI – OPH_3	-8.2	-9.3	-6.9	-5.7	-2.9	8.4	-7.4
MeI – OPH_3	-3.8	-4.0	-4.5	-5.1	-2.7	-5.4	-5.7
NCH – NBS	-3.5	-3.4	-2.8	-2.8	-1.4	-1.9	-3.5
NCH – NIS	-4.3	-4.3	-3.7	-3.5	-1.7	3.7	-4.3

Table S6: continued from previous page.

Dimer	$E^{(2)}_{\rm dispx}$	$D^{20}_{\rm as}$	D3BJ(HF)	D3BJ(OLYP)	D3BJ(revPBE38)	D3(NS)	DD3S
NH3 – NBS	-5.5	-5.2	-3.9	-3.9	-1.8	-3.0	-4.8
NH3 – NIS	-6.8	-6.8	-5.3	-4.8	-2.3	-5.1	-6.1
PCH – NBS	-2.6	-2.1	-2.9	-3.1	-1.7	-3.5	-3.5
PCH – NIS	-3.1	-2.6	-3.7	-3.6	-2.0	-4.5	-4.2
MUE/MURE:		0.4/10.3	0.9/18.8	1.0/20.0	2.4/51.9	5.5/89.1	0.7/17.5

[a]In units of kcal · mol^{-1}.

[b]Benchmark reported in Ref. S8.

[c]$D_{\rm as}$ revision reported herein.

[d]D3[S19] dispersion term calculated with BJ damping for the HF level of theory or given functional.

[e]D3[S19] dispersion term calculated without switching.[S20]

[f]Method reported in Ref. S21.

Table S7: $E^{(2)}_{\rm dispx}$, $D^{20}_{\rm as}$, $D^{10}_{\rm as}$, D3, and DD3S energies[a] calculated for each dimer from the NBC10ext database[b]. Corresponding MUE and MURE values are given.

Dimer	R/R$_{eq}$	$E^{(2)}_{\rm dispx}$	$D^{20\,c}_{\rm as}$	$D^{10\,d}_{\rm as}$	D3BJ(HF)[e]	D3BJ(OLYP)[e]	D3BJ(revPBE38)[e]	D3(NS)[f]	DD3S[g]
**1** (C_6H_6 dimer)	0.82	-12.5	-12.7	-13.4	-11.4	-15.8	-9.5	-8.1	-15.4
	0.85	-10.8	-10.8	-11.4	-9.7	-13.7	-8.5	-7.1	-13.2
	0.87	-9.3	-9.3	-9.7	-8.4	-11.9	-7.6	-6.2	-11.2
	0.90	-8.0	-7.9	-8.3	-7.2	-10.3	-6.7	-5.4	-9.6
	0.92	-7.0	-6.8	-7.1	-6.2	-8.9	-6.0	-4.7	-8.3
	0.95	-6.0	-5.9	-6.1	-5.4	-7.7	-5.3	-4.1	-7.1
	0.97	-5.2	-5.1	-5.3	-4.7	-6.7	-4.7	-3.6	-6.1
	1.00	-4.6	-4.4	-4.5	-4.1	-5.8	-4.1	-3.2	-5.3
	1.03	-4.0	-3.8	-3.9	-3.6	-5.0	-3.7	-2.8	-4.6
	1.05	-3.5	-3.3	-3.4	-3.1	-4.4	-3.2	-2.4	-4.0
	1.08	-3.0	-2.9	-3.0	-2.7	-3.8	-2.9	-2.1	-3.5
	1.15	-2.0	-1.9	-2.0	-1.9	-2.6	-2.0	-1.5	-2.3
	1.28	-1.1	-1.0	-1.1	-1.1	-1.4	-1.1	-0.8	-1.3
	1.41	-0.6	-0.6	-0.6	-0.6	-0.8	-0.7	-0.5	-0.7
	1.54	-0.4	-0.4	-0.4	-0.4	-0.5	-0.4	-0.3	-0.4
	1.67	-0.2	-0.2	-0.2	-0.2	-0.3	-0.3	-0.2	-0.3
	2.56	0.0	0.0	0.0	0.0	0.0	0.0	0.0	0.0
**2** (C_6H_6 dimer)	0.86	-11.0	-11.4	-12.3	-11.2	-13.0	-6.7	-13.1	-15.2
	0.87	-10.2	-10.6	-11.4	-10.4	-12.3	-6.4	-11.9	-14.2
	0.88	-9.5	-9.8	-10.5	-9.7	-11.6	-6.1	-10.8	-13.2
	0.90	-8.2	-8.4	-9.0	-8.4	-10.3	-5.6	-9.0	-11.5
	0.92	-7.0	-7.2	-7.7	-7.3	-9.2	-5.1	-7.5	-9.9
	0.94	-6.1	-6.2	-6.6	-6.3	-8.1	-4.6	-6.3	-8.5
	0.96	-5.2	-5.3	-5.6	-5.4	-7.1	-4.1	-5.2	-7.3
	0.98	-4.5	-4.6	-4.8	-4.7	-6.2	-3.7	-4.4	-6.2
	1.00	-3.9	-3.9	-4.1	-4.0	-5.4	-3.3	-3.7	-5.3

Table S7: continued from previous page.

Dimer	R/R$_{eq}$	$E^{(2)}_{\text{dispx}}$	D^{20}_{as}	D^{10}_{as}	D3BJ(HF)	D3BJ(OLYP)	D3BJ(revPBE38)	D3(NS)	DD3S
**2** (C_6H_6 dimer)	1.02	-3.4	-3.4	-3.6	-3.5	-4.7	-3.0	-3.2	-4.6
	1.04	-3.0	-2.9	-3.1	-3.0	-4.1	-2.7	-2.7	-3.9
	1.06	-2.6	-2.5	-2.7	-2.6	-3.6	-2.4	-2.3	-3.4
	1.08	-2.2	-2.2	-2.3	-2.2	-3.1	-2.1	-2.0	-2.9
	1.10	-2.0	-1.9	-2.0	-2.0	-2.7	-1.9	-1.7	-2.5
	1.12	-1.7	-1.7	-1.7	-1.7	-2.3	-1.7	-1.5	-2.2
	1.20	-1.0	-1.0	-1.0	-1.0	-1.4	-1.0	-0.8	-1.2
	1.30	-0.6	-0.5	-0.6	-0.6	-0.7	-0.6	-0.4	-0.7
	1.40	-0.3	-0.3	-0.3	-0.3	-0.4	-0.3	-0.2	-0.4
	1.50	-0.2	-0.2	-0.2	-0.2	-0.2	-0.2	-0.1	-0.2
	1.60	-0.1	-0.1	-0.1	-0.1	-0.2	-0.1	-0.1	-0.1
**3** (C_6H_6 dimer)	0.75	-9.3	-9.2	-9.6	-8.3	-11.8	-7.6	-6.2	-11.2
	0.78	-9.2	-9.1	-9.5	-8.3	-11.7	-7.5	-6.1	-11.1
	0.81	-9.0	-9.0	-9.4	-8.1	-11.5	-7.4	-6.1	-10.9
	0.84	-8.8	-8.7	-9.1	-7.9	-11.3	-7.2	-5.9	-10.6
	0.88	-8.5	-8.5	-8.8	-7.7	-10.9	-7.1	-5.8	-10.3
	0.91	-8.2	-8.1	-8.5	-7.4	-10.5	-6.8	-5.6	-9.9
	0.94	-7.8	-7.8	-8.1	-7.1	-10.1	-6.6	-5.4	-9.5
	0.95	-7.6	-7.6	-7.9	-7.0	-9.9	-6.4	-5.3	-9.3
	0.97	-7.4	-7.4	-7.7	-6.8	-9.6	-6.3	-5.2	-9.1
	0.98	-7.2	-7.2	-7.5	-6.6	-9.4	-6.1	-5.1	-8.8
	1.00	-7.0	-7.0	-7.3	-6.4	-9.1	-6.0	-4.9	-8.6
	1.06	-6.8	-6.8	-7.1	-6.3	-8.9	-5.8	-4.8	-8.3
	1.11	-6.6	-6.6	-6.9	-6.1	-8.6	-5.7	-4.7	-8.1
	1.22	-6.2	-6.1	-6.4	-5.7	-8.0	-5.3	-4.4	-7.6
	1.33	-5.8	-5.7	-5.9	-5.3	-7.5	-5.0	-4.1	-7.0
	1.44	-5.3	-5.2	-5.5	-4.9	-6.9	-4.6	-3.8	-6.5
	1.56	-4.9	-4.8	-5.0	-4.5	-6.4	-4.3	-3.6	-6.0
	1.67	-4.5	-4.4	-4.5	-4.1	-5.8	-3.9	-3.3	-5.4
**4** ($C_6H_6 - SH_2$)	0.83	-10.3	-11.4	-13.1	-10.3	-12.4	-6.1	-11.0	-14.2
	0.84	-9.5	-10.5	-12.0	-9.5	-11.7	-5.8	-10.0	-13.2
	0.87	-8.1	-8.9	-10.2	-8.2	-10.4	-5.3	-8.3	-11.3
	0.89	-7.0	-7.6	-8.7	-7.0	-9.2	-4.9	-6.9	-9.7
	0.92	-6.0	-6.5	-7.4	-6.0	-8.0	-4.4	-5.8	-8.2
	0.95	-5.1	-5.6	-6.3	-5.1	-7.0	-4.0	-4.9	-7.0
	0.97	-4.4	-4.8	-5.3	-4.3	-6.1	-3.6	-4.1	-5.9
	1.00	-3.8	-4.1	-4.6	-3.7	-5.2	-3.2	-3.5	-5.1
	1.03	-3.2	-3.5	-3.9	-3.2	-4.5	-2.8	-3.0	-4.3
	1.05	-2.8	-3.0	-3.3	-2.7	-3.9	-2.5	-2.5	-3.7
	1.08	-2.4	-2.6	-2.9	-2.3	-3.4	-2.2	-2.2	-3.1
	1.11	-2.1	-2.3	-2.5	-2.0	-2.9	-2.0	-1.9	-2.7
	1.18	-1.3	-1.5	-1.6	-1.3	-1.9	-1.4	-1.2	-1.7

Table S7: continued from previous page.

Dimer	R/R$_{eq}$	$E^{(2)}_{\text{dispx}}$	D^{20}_{as}	D^{10}_{as}	D3BJ(HF)	D3BJ(OLYP)	D3BJ(revPBE38)	D3(NS)	DD3S
**4** (C_6H_6 – SH_2)	1.25	-1.0	-1.1	-1.1	-0.9	-1.3	-1.0	-0.9	-1.2
	1.32	-0.7	-0.8	-0.8	-0.7	-1.0	-0.7	-0.6	-0.9
	1.38	-0.5	-0.6	-0.6	-0.5	-0.7	-0.5	-0.5	-0.6
	1.45	-0.4	-0.4	-0.4	-0.4	-0.5	-0.4	-0.3	-0.5
	1.58	-0.2	-0.2	-0.2	-0.2	-0.3	-0.2	-0.2	-0.3
	1.71	-0.1	-0.1	-0.1	-0.1	-0.2	-0.1	-0.1	-0.2
	1.84	-0.1	-0.1	-0.1	-0.1	-0.1	-0.1	-0.1	-0.1
	1.97	0.0	-0.1	-0.1	-0.1	-0.1	-0.1	0.0	-0.1
**5** (C_6H_6 – CH_4)	0.83	-6.7	-6.2	-6.9	-6.4	-7.7	-3.8	-7.7	-8.8
	0.84	-6.2	-5.8	-6.4	-6.0	-7.2	-3.7	-7.0	-8.2
	0.87	-5.3	-4.9	-5.4	-5.1	-6.4	-3.3	-5.8	-7.0
	0.89	-4.6	-4.2	-4.6	-4.4	-5.6	-3.0	-4.8	-6.0
	0.92	-3.9	-3.6	-4.0	-3.8	-4.9	-2.7	-4.0	-5.1
	0.95	-3.4	-3.1	-3.4	-3.2	-4.3	-2.5	-3.3	-4.4
	0.97	-2.9	-2.6	-2.9	-2.8	-3.7	-2.2	-2.8	-3.7
	1.00	-2.5	-2.3	-2.5	-2.4	-3.2	-2.0	-2.4	-3.2
	1.03	-2.2	-1.9	-2.1	-2.0	-2.8	-1.8	-2.0	-2.7
	1.05	-1.9	-1.7	-1.8	-1.7	-2.4	-1.6	-1.7	-2.3
	1.08	-1.6	-1.5	-1.6	-1.5	-2.1	-1.4	-1.4	-2.0
	1.11	-1.4	-1.3	-1.4	-1.3	-1.8	-1.2	-1.2	-1.7
	1.16	-1.1	-0.9	-1.0	-1.0	-1.3	-1.0	-0.9	-1.2
	1.21	-0.8	-0.7	-0.8	-0.7	-1.0	-0.8	-0.7	-0.9
	1.26	-0.6	-0.5	-0.6	-0.6	-0.8	-0.6	-0.5	-0.7
	1.32	-0.5	-0.4	-0.4	-0.4	-0.6	-0.5	-0.4	-0.5
	1.37	-0.4	-0.3	-0.3	-0.3	-0.5	-0.4	-0.3	-0.4
	1.42	-0.3	-0.3	-0.3	-0.3	-0.4	-0.3	-0.2	-0.3
	1.47	-0.2	-0.2	-0.2	-0.2	-0.3	-0.2	-0.2	-0.3
	1.58	-0.1	-0.1	-0.1	-0.1	-0.2	-0.2	-0.1	-0.2
**6** (CH_4 dimer)	0.86	-3.0	-2.8	-2.9	-3.0	-3.7	-1.8	-3.3	-4.1
	0.88	-2.7	-2.6	-2.7	-2.7	-3.5	-1.7	-3.0	-3.7
	0.89	-2.5	-2.3	-2.4	-2.5	-3.2	-1.6	-2.7	-3.4
	0.92	-2.1	-2.0	-2.0	-2.1	-2.7	-1.4	-2.2	-2.8
	0.94	-1.8	-1.7	-1.7	-1.7	-2.3	-1.3	-1.8	-2.3
	0.97	-1.5	-1.4	-1.4	-1.5	-2.0	-1.2	-1.5	-1.9
	1.00	-1.2	-1.2	-1.2	-1.2	-1.7	-1.0	-1.2	-1.6
	1.03	-1.1	-1.0	-1.0	-1.0	-1.4	-0.9	-1.0	-1.3
	1.06	-0.9	-0.8	-0.9	-0.9	-1.2	-0.8	-0.9	-1.1
	1.08	-0.8	-0.7	-0.7	-0.7	-1.0	-0.7	-0.7	-1.0
	1.11	-0.6	-0.6	-0.6	-0.6	-0.9	-0.6	-0.6	-0.8
	1.14	-0.5	-0.5	-0.5	-0.5	-0.7	-0.5	-0.5	-0.7
	1.17	-0.5	-0.5	-0.5	-0.5	-0.6	-0.5	-0.4	-0.6
	1.19	-0.4	-0.4	-0.4	-0.4	-0.5	-0.4	-0.4	-0.5

Table S7: continued from previous page.

Dimer	R/R$_{eq}$	$E^{(2)}_{\mathrm{dispx}}$	D^{20}_{as}	D^{10}_{as}	D3BJ(HF)	D3BJ(OLYP)	D3BJ(revPBE38)	D3(NS)	DD3S
**6** (CH_4 dimer)	1.22	-0.3	-0.3	-0.3	-0.3	-0.5	-0.3	-0.3	-0.4
	1.28	-0.3	-0.3	-0.3	-0.3	-0.3	-0.3	-0.2	-0.3
	1.33	-0.2	-0.2	-0.2	-0.2	-0.3	-0.2	-0.2	-0.2
	1.39	-0.1	-0.1	-0.1	-0.2	-0.2	-0.2	-0.1	-0.2
	1.50	-0.1	-0.1	-0.1	-0.1	-0.1	-0.1	-0.1	-0.1
	1.61	-0.1	-0.1	-0.1	-0.1	-0.1	-0.1	-0.1	-0.1
**7** (pyridine dimer)	0.84	-13.1	-13.9	-14.8	-12.1	-16.6	-9.6	-7.8	-16.5
	0.86	-11.2	-11.8	-12.5	-10.3	-14.4	-8.7	-7.0	-14.0
	0.89	-9.6	-10.1	-10.6	-8.9	-12.5	-7.7	-6.2	-12.0
	0.92	-8.3	-8.6	-9.0	-7.6	-10.8	-6.9	-5.4	-10.2
	0.95	-7.1	-7.4	-7.7	-6.5	-9.3	-6.1	-4.7	-8.7
	0.97	-6.2	-6.3	-6.6	-5.6	-8.1	-5.4	-4.1	-7.5
	1.00	-5.3	-5.4	-5.6	-4.9	-7.0	-4.8	-3.6	-6.4
	1.03	-4.6	-4.7	-4.9	-4.2	-6.0	-4.3	-3.2	-5.5
	1.05	-4.0	-4.1	-4.2	-3.7	-5.2	-3.8	-2.8	-4.8
	1.08	-3.5	-3.5	-3.6	-3.2	-4.5	-3.3	-2.4	-4.2
	1.11	-3.0	-3.1	-3.2	-2.8	-4.0	-2.9	-2.1	-3.6
	1.14	-2.7	-2.7	-2.8	-2.5	-3.5	-2.6	-1.9	-3.1
	1.16	-2.3	-2.3	-2.4	-2.2	-3.0	-2.3	-1.6	-2.7
	1.19	-2.0	-2.0	-2.1	-1.9	-2.6	-2.0	-1.5	-2.4
	1.22	-1.8	-1.8	-1.9	-1.7	-2.3	-1.8	-1.3	-2.1
	1.27	-1.4	-1.4	-1.4	-1.3	-1.8	-1.4	-1.0	-1.6
	1.35	-1.0	-1.0	-1.0	-0.9	-1.3	-1.0	-0.7	-1.2
	1.49	-0.5	-0.6	-0.6	-0.6	-0.7	-0.6	-0.4	-0.7
	1.62	-0.3	-0.3	-0.3	-0.3	-0.4	-0.4	-0.2	-0.4
	1.76	-0.2	-0.2	-0.2	-0.2	-0.3	-0.2	-0.1	-0.2
	1.89	-0.1	-0.1	-0.1	-0.1	-0.2	-0.2	-0.1	-0.2
**8** (pyridine dimer)	0.84	-13.7	-14.8	-15.5	-13.0	-15.0	-7.4	-16.4	-17.8
	0.87	-10.4	-11.1	-11.6	-10.3	-12.4	-6.4	-11.4	-14.0
	0.88	-9.9	-10.5	-11.0	-9.8	-11.9	-6.2	-10.7	-13.4
	0.89	-9.0	-9.5	-9.9	-9.0	-11.1	-5.9	-9.4	-12.3
	0.91	-7.9	-8.3	-8.7	-7.9	-10.0	-5.4	-8.0	-10.9
	0.92	-7.2	-7.6	-7.9	-7.2	-9.3	-5.1	-7.2	-9.9
	0.94	-6.2	-6.4	-6.7	-6.2	-8.1	-4.6	-5.9	-8.4
	0.96	-5.3	-5.5	-5.7	-5.3	-7.1	-4.2	-4.9	-7.2
	0.98	-4.5	-4.7	-4.8	-4.5	-6.2	-3.8	-4.1	-6.1
	1.00	-3.9	-4.0	-4.1	-3.9	-5.3	-3.4	-3.5	-5.2
	1.02	-3.4	-3.4	-3.5	-3.4	-4.6	-3.0	-2.9	-4.4
	1.04	-2.9	-3.0	-3.0	-2.9	-4.0	-2.7	-2.5	-3.8
	1.06	-2.5	-2.6	-2.6	-2.5	-3.5	-2.4	-2.1	-3.2
	1.08	-2.2	-2.2	-2.3	-2.2	-3.0	-2.1	-1.8	-2.8
	1.10	-1.9	-1.9	-2.0	-1.9	-2.6	-1.9	-1.6	-2.4

Table S7: continued from previous page.

Dimer	R/R$_{eq}$	$E^{(2)}_{\text{dispx}}$	D^{20}_{as}	D^{10}_{as}	D3BJ(HF)	D3BJ(OLYP)	D3BJ(revPBE38)	D3(NS)	DD3S
**8** (pyridine dimer)	1.12	-1.6	-1.7	-1.7	-1.6	-2.2	-1.6	-1.4	-2.1
	1.16	-1.3	-1.3	-1.3	-1.3	-1.7	-1.3	-1.0	-1.6
	1.22	-0.9	-0.9	-0.9	-0.9	-1.1	-0.9	-0.7	-1.1
	1.33	-0.5	-0.5	-0.5	-0.5	-0.6	-0.5	-0.4	-0.6
	1.43	-0.3	-0.3	-0.3	-0.3	-0.4	-0.3	-0.2	-0.3
	1.63	-0.1	-0.1	-0.1	-0.1	-0.1	-0.1	-0.1	-0.1
	1.84	0.0	0.0	0.0	-0.1	-0.1	-0.1	0.0	-0.1
**9** (C_6H_6 dimer)	0.75	-12.5	-12.6	-13.3	-11.3	-15.7	-9.5	-8.0	-15.4
	0.78	-12.3	-12.5	-13.1	-11.2	-15.6	-9.4	-8.0	-15.2
	0.81	-12.1	-12.2	-12.9	-11.0	-15.3	-9.3	-7.9	-14.9
	0.84	-11.8	-11.9	-12.5	-10.7	-15.0	-9.1	-7.7	-14.5
	0.87	-11.4	-11.5	-12.1	-10.4	-14.5	-8.8	-7.6	-14.1
	0.90	-10.9	-11.1	-11.6	-10.0	-14.0	-8.6	-7.3	-13.6
	0.93	-10.4	-10.6	-11.1	-9.6	-13.4	-8.2	-7.1	-13.0
	0.94	-10.2	-10.3	-10.8	-9.4	-13.1	-8.1	-6.9	-12.7
	0.96	-9.9	-10.0	-10.6	-9.1	-12.8	-7.9	-6.8	-12.3
	0.97	-9.6	-9.7	-10.3	-8.9	-12.5	-7.7	-6.6	-12.0
	0.99	-9.4	-9.5	-10.0	-8.6	-12.1	-7.5	-6.4	-11.7
	1.00	-9.1	-9.2	-9.6	-8.4	-11.7	-7.3	-6.3	-11.3
	1.05	-8.8	-8.9	-9.3	-8.1	-11.4	-7.1	-6.1	-10.9
	1.16	-8.2	-8.3	-8.7	-7.6	-10.6	-6.6	-5.7	-10.2
	1.26	-7.7	-7.6	-8.0	-7.1	-9.9	-6.2	-5.4	-9.5
	1.37	-7.1	-7.0	-7.4	-6.5	-9.1	-5.8	-5.0	-8.7
	1.47	-6.5	-6.4	-6.7	-6.0	-8.4	-5.3	-4.6	-8.0
	1.58	-5.9	-5.8	-6.1	-5.5	-7.6	-4.9	-4.3	-7.3
**10** (C_6H_6 dimer)	0.75	-6.9	-6.8	-7.1	-6.2	-8.9	-6.0	-4.7	-8.2
	0.78	-6.9	-6.7	-7.0	-6.1	-8.8	-5.9	-4.7	-8.1
	0.82	-6.7	-6.6	-6.9	-6.0	-8.6	-5.8	-4.6	-8.0
	0.85	-6.6	-6.5	-6.7	-5.9	-8.4	-5.7	-4.5	-7.8
	0.88	-6.4	-6.3	-6.5	-5.7	-8.2	-5.6	-4.4	-7.6
	0.92	-6.1	-6.0	-6.3	-5.5	-7.9	-5.4	-4.3	-7.3
	0.95	-5.9	-5.8	-6.0	-5.3	-7.6	-5.2	-4.1	-7.0
	0.97	-5.7	-5.6	-5.9	-5.2	-7.4	-5.1	-4.0	-6.9
	0.98	-5.6	-5.5	-5.7	-5.1	-7.2	-5.0	-3.9	-6.7
	1.00	-5.5	-5.4	-5.6	-5.0	-7.1	-4.9	-3.9	-6.5
	1.06	-5.3	-5.2	-5.4	-4.8	-6.9	-4.7	-3.8	-6.4
	1.12	-5.2	-5.1	-5.3	-4.7	-6.7	-4.6	-3.7	-6.2
	1.18	-5.0	-4.9	-5.1	-4.6	-6.5	-4.5	-3.6	-6.0
	1.29	-4.7	-4.6	-4.8	-4.3	-6.1	-4.2	-3.4	-5.6
	1.41	-4.4	-4.3	-4.4	-4.0	-5.7	-3.9	-3.2	-5.2
	1.53	-4.1	-3.9	-4.1	-3.7	-5.3	-3.7	-2.9	-4.9
	1.65	-3.7	-3.6	-3.8	-3.4	-4.8	-3.4	-2.7	-4.5

Table S7: continued from previous page.

Dimer	R/R$_{eq}$	$E^{(2)}_{\text{dispx}}$	D^{20}_{as}	D^{10}_{as}	D3BJ(HF)	D3BJ(OLYP)	D3BJ(revPBE38)	D3(NS)	DD3S
	1.76	-3.4	-3.3	-3.4	-3.2	-4.4	-3.1	-2.5	-4.1
MUE/MURE:			0.1/4.1	0.3/6.0	0.3/5.3	1.3/30.0	0.9/15.3	1.1/20.3	1.2/25.0

[a]In units of kcal · mol^{-1}.
[b]Benchmark reported in Ref. S7
[c]D_{as} revision reported herein.
[d]D_{as} revision reported in Ref. S22.
[e]D3[S19] dispersion term calculated with BJ damping for the HF level of theory or given functional.
[f]D3[S19] dispersion term calculated without switching.[S20]
[g]Method reported in Ref. S21.

Table S8: MP2 and MED interaction energy with its $E^{(10)}_{\text{EL,MTP}}$ and D^{20}_{as} contributions[a], computed for uPA inhibitors.

Inhibitor[b]	pIC_{50}[b]	$E^{(10)}_{\text{EL,MTP}}$	D^{20}_{as}	MED	MP2[c]
**1**	8.19	-157.6	-69.1	-226.7	-135.8
**2**	6.27	-129.8	-55.6	-185.4	-122.6
**3**	6.12	-149.9	-56.4	-206.3	-133.2
**4**	6.11	-130.3	-50.8	-181.1	-124.0
**5**	3.80	-102.1	-37.2	-139.3	-119.6

[a]In units of kcal · mol^{-1}.
[b]Numbering of the inhibitors is consistent with Ref. S23, from which the pIC_{50} values were taken.
[c]Values were provided by Grzywa et al.[S23].

Table S9: MP2 and MED interaction energy with its $E^{(10)}_{\text{EL,MTP}}$ and D^{20}_{as} contributions[a], computed for HB alcohol dimers.

Dimer[b]	SAPT[b]	MP2	$E^{(10)}_{\text{EL,MTP}}$	D^{20}_{as}	MED
**H_2O**	-4.6	-3.2	-4.9	-2.3	-7.1
**MeOH**	-5.4	-5.5	-7.1	-4.8	-11.8
**EtOH**	-5.6	-5.7	-6.8	-5.3	-12.0
**nPrOH**	-5.9	-5.9	-6.3	-5.4	-11.7
**nBuOH**	-6.0	-5.9	-5.8	-5.5	-11.4
**iPrOH**	-6.5	-6.5	-5.9	-6.9	-12.7
**tBuOH**	-7.2	-7.3	-7.7	-7.6	-15.4

[a]In units of kcal · mol^{-1}.
[b]Naming of dimers is consistent with Ref. S24, from which the reference SAPT energy values were taken.

Table S10: Performance of the selected empirical scoring functions[a] for the ranking of uPA inhibitors.

Inhibitor[b]	$PLANTS_{PLP}$	RankScore	$PLANTS_{CHEMPLP}$	Vina	DSX	ChemScore	ChemPLP	ASP	AutoDock	GoldScore
**1**	-45.5	0.0	-53.9	-1.5	-38.8	9.4	25.3	15.4	-2.4	36.5
**2**	-47.2	-17.4	-56.1	-1.9	-44.9	14.1	29.2	24.9	-1.7	44.5
**3**	-44.8	-12.6	-54.4	-0.5	-32.0	13.0	25.0	9.8	3.1	35.1
**4**	-48.1	2.2	-46.1	0.2	-25.2	1.6	5.7	11.1	-1.6	6.0
**5**	-58.0	-31.8	-66.2	-4.5	-60.1	18.2	41.4	21.6	-4.6	37.2
R[c]	0.86	0.81	0.63	0.61	0.58	0.50	0.46	0.32	0.31	0.01
R^2	0.74	0.66	0.40	0.37	0.34	0.25	0.21	0.10	0.10	0.00
N_{pred}[d]	20.0	40.0	40.0	50.0	50.0	40.0	50.0	50.0	50.0	60.0

[a]The scores obtained with particular scoring functions are given in units of $kcal \cdot mol^{-1}$ ($PLANTS_{PLP}$, $PLANTS_{CHEMPLP}$, Vina, AutoDock) or in arbitrary units (GoldScore, ChemScore, ChemPLP, ASP, DSX, RankScore).

[b]Numbering of the inhibitors is consistent with Ref. S23.

[c]Correlation coefficient between the energy obtained at a given level of theory and the experimental inhibitory activity (expressed as pIC_{50} values) taken from Ref. S23. In the case of GoldScore, ChemScore, ChemPLP and ASP functions, for which higher score indicates greater inhibitory activity, the opposite of the correlation coefficient value is given to facilitate direct comparison with the results of the remaining empirical scoring functions (or the MED model), wherein the more potent inhibitor is associated with a lower score (or the binding energy) value.

[d]Percentage of successful predictions [%].

Table S11: Differential intermediate state stabilization[a] in ketosteroid isomerase-catalyzed reaction.[b]

Residue	$E^{(10)}_{\mathrm{EL,MTP}}$	D^{20}_{as}	MED
**TYR14**	-7.7	-0.7	-8.5
**LEU18**	-0.2	-0.1	-0.3
**PRO39**	0.3	0.0	0.3
**PHE54**	0.4	0.4	0.8
**TYR55**	-1.6	0.0	-1.7
**SER58**	0.8	0.1	0.9
**LEU59**	0.0	0.0	0.0
**LEU61**	0.2	0.0	0.2
**LEU63**	0.0	0.0	0.0
**VAL65**	-0.2	0.0	-0.2
**PHE80**	0.1	0.0	0.1
**PHE82**	-1.0	-0.1	-1.1
**VAL84**	-0.1	-0.1	-0.2
**PHE86**	-0.2	0.2	0.1
**THR93**	0.2	0.0	0.2
**VAL95**	0.5	0.0	0.5
**PRO97**	-0.3	-0.1	-0.4
**ASP99**	-6.4	-0.5	-7.0
**MET112**	-2.1	-0.4	-2.5
**ALA114**	-1.0	-0.5	-1.5
**PHE116**	1.2	-0.2	1.0
**ILE121**	-0.1	0.0	-0.1
**total**	-17.3	-2.1	-19.4

[a] In units of $\mathrm{kcal \cdot mol^{-1}}$.

[b] The structures of enzyme-intermediate and enzyme-substrate complexes of *C. testosteroni* ketosteroid isomerase were derived from the QM/MM simulation reported in Ref. S25.

Table S12: Differential intermediate state stabilization[a] in ketosteroid isomerase-catalyzed reaction — amino acid residue rotamers optimized with respect to DISS lowering. For comparison with the total DISS value characterizing the KSI structures reported in Ref. S25, the respective DISS contributions of Pro39, Pro97, and Ala114 residues are included without rotamer modification.

Residue	$E^{(10)}_{\mathrm{EL,MTP}}$	D^{20}_{as}	MED
**TYR14**	-5.7	-0.5	-6.2
**LEU18**	-0.4	0.0	-0.4
**PRO39[b]**	0.3	-0.0	0.3
**PHE54**	0.0	0.0	0.0
**TYR55**	-0.9	0.0	-0.9
**SER58**	-0.3	0.1	-0.2
**LEU59**	0.0	0.0	0.0
**LEU61**	0.0	0.0	0.0
**LEU63**	-0.1	0.0	0.0
**VAL65**	-0.2	0.0	-0.2
**PHE80**	-0.4	0.0	-0.4
**PHE82**	-1.5	-0.2	-1.8
**VAL84**	-0.8	-0.1	-0.8
**PHE86**	-0.3	0.2	-0.1
**THR93**	0.0	0.0	0.0
**VAL95**	-0.4	0.0	-0.4
**PRO97[b]**	-0.3	-0.1	-0.4
**ASH99**	-6.5	-0.4	-6.9
**MET112**	-2.0	-0.2	-2.2
**ALA114[b]**	-1.0	-0.5	-1.5
**PHE116**	1.2	-0.1	1.1
**ILE121**	0.1	0.0	0.1
**total**	-19.0	-1.8	-20.8

[a] In units of $\mathrm{kcal \cdot mol^{-1}}$.

[b] The structures of enzyme-intermediate and enzyme-substrate complexes of *C. testosteroni* ketosteroid isomerase were derived from the QM/MM simulation reported in Ref. S25.

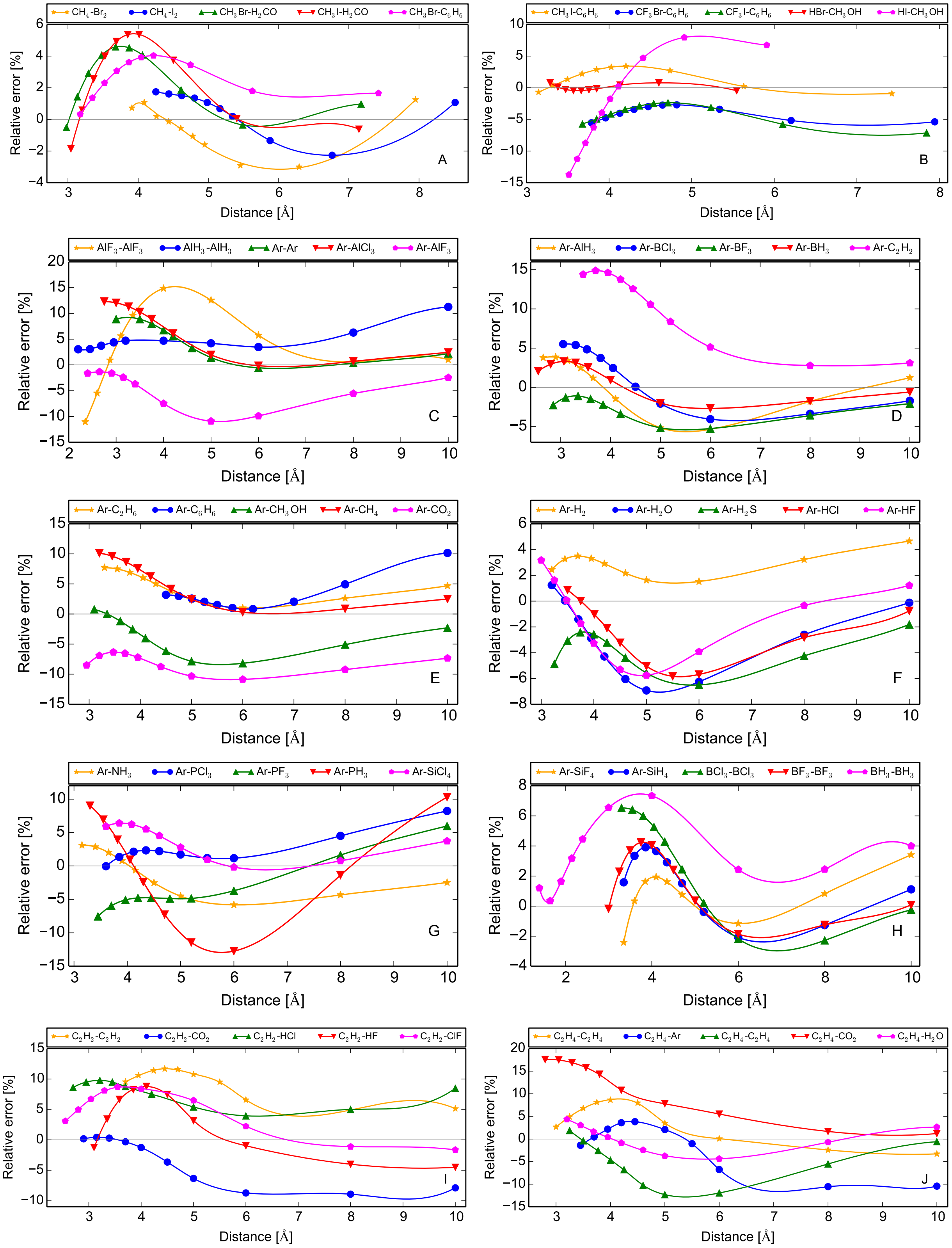

Figure S1: Signed relative errors (in percent) of the new D_{as}^{20} function with respect to the benchmark $E_{\mathrm{dispx}}^{(2)}$ for all dimers from the training set.

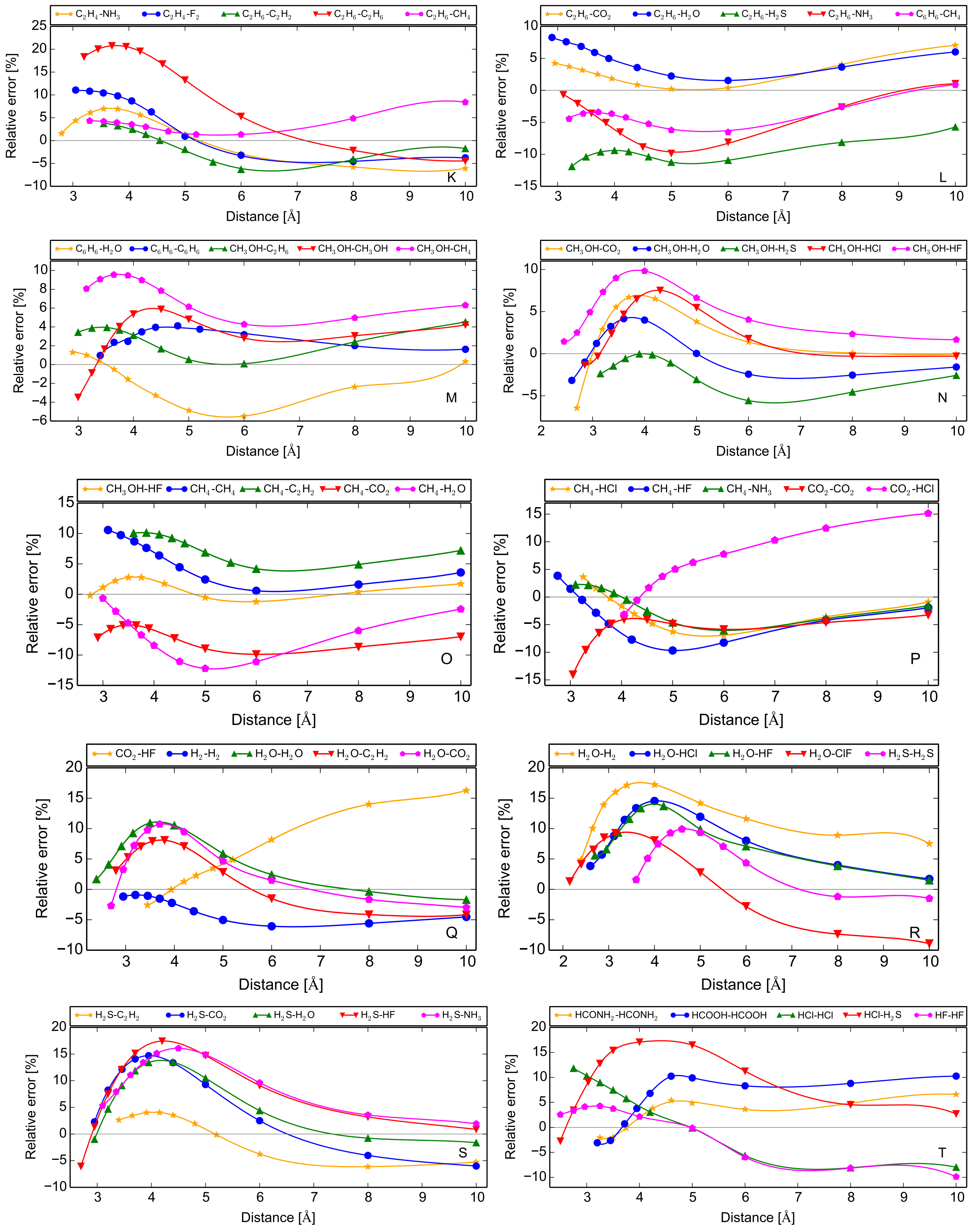

Figure S1: continued from previous page.

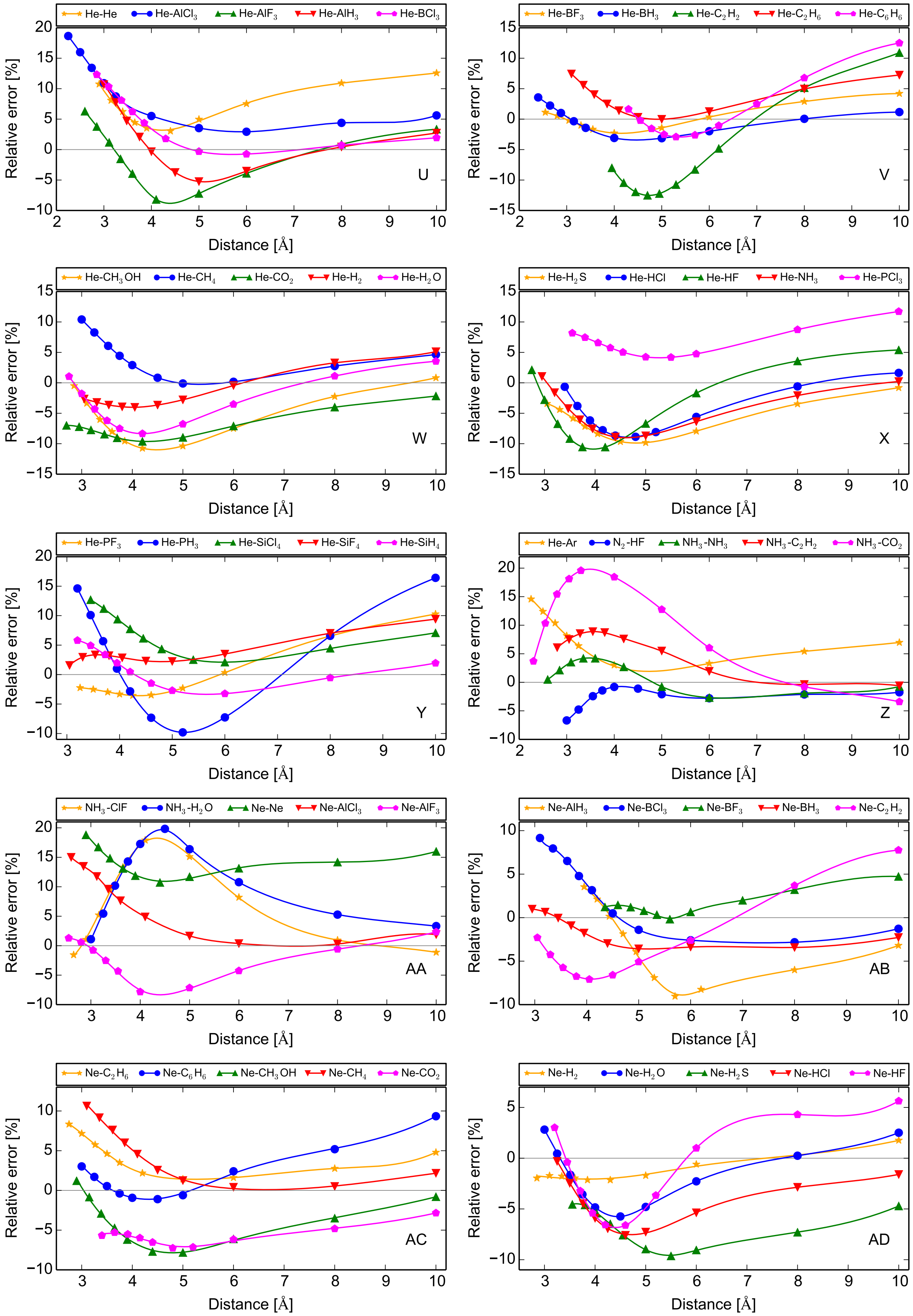

Figure S1: continued from previous page.

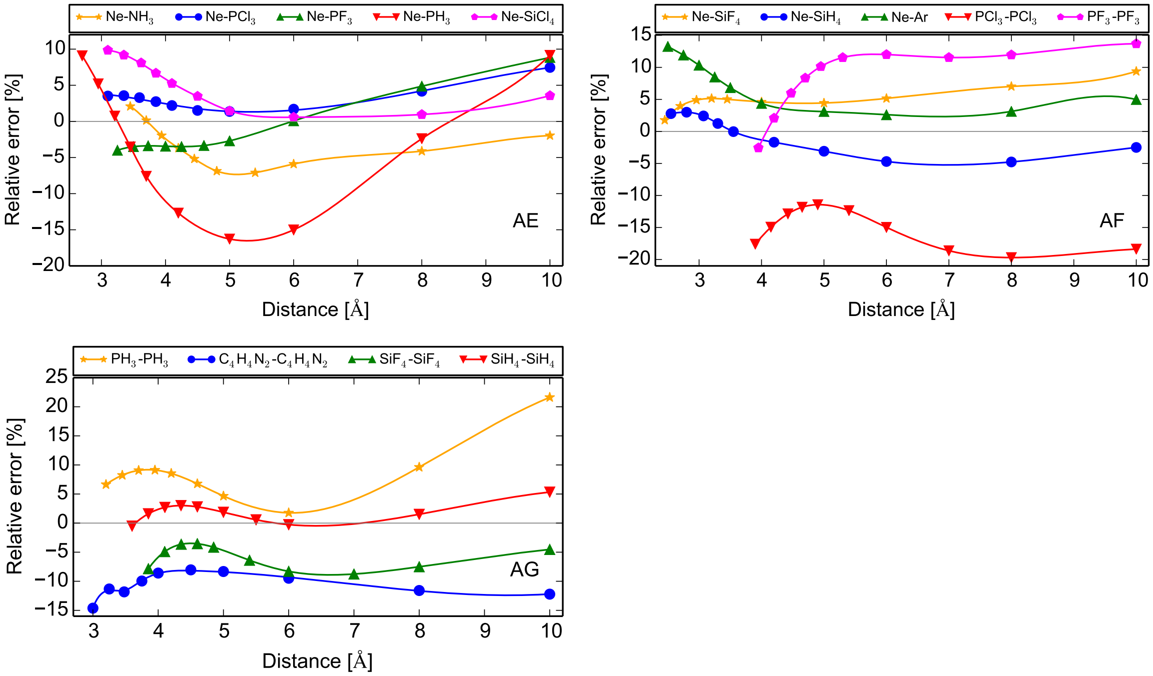

Figure S1: continued from previous page.

References

(S1) Bukowski, R.; Cencek, W.; Jankowski, P.; Jeziorska, M.; Jeziorski, B.; Kucharski, S.; Lotrich, V.; Misquitta, A.; Moszynski, K., R. Patkowski; Podeszwa, R.; Rybak, S.; Szalewicz, K.; Williams, H.; Wheatley, R.; Wormer, P.; Żuchowski, P. SAPT2008: An ab initio program for many-body symmetry-adapted perturbation theory calculations of intermolecular interaction energies. 2016; `http://www.physics.udel.edu/szalewic/SAPT/SAPT.html`, Version 2008.2, University of Delaware and University of Warsaw.

(S2) Perdew, J. P.; Emzerhof, M.; Burke, K. Generalized gradient approximation made simple. *Phys. Rev. Lett.* **1996**, *77*, 3865–3868.

(S3) Adamo, C.; Barone, V. Toward reliable density functional methods without adjustable parameters: The PBE0 model. *J. Chem. Phys.* **1999**, *110*, 6158–6170.

(S4) Misquitta, A. J.; Szalewicz, K. Symmetry-adapted perturbation-theory calculations of intermolecular forces employing density-functional description of monomers. *J. Chem. Phys.* **2005**, *122*.

(S5) Kendall, R. A.; Dunning, T. H.; Harrison, R. J. Electron affinities of the first-row atoms revisited. Systematic basis sets and wave functions. *J. Chem. Phys.* **1992**, *96*, 6796–6806.

(S6) Rezac, J.; Riley, K. E.; Hobza, P. Benchmark calculations of noncovalent interactions of halogenated molecules. *J. Chem. Theory Comput.* **2012**, *8*, 4285–4292.

(S7) Smith, D. G.; Burns, L. A.; Patkowski, K.; Sherrill, C. D. Revised damping parameters for the D3 dispersion correction to density functional theory. *J. Phys. Chem. Lett.* **2016**, *7*, 2197–2203.

(S8) Kozuch, S.; Martin, J. M. L. Halogen bonds: Benchmarks and theoretical analysis. *J. Chem. Theory Comput.* **2013**, *9*, 1918–1931.

(S9) Hesselmann, A.; Jansen, G. Intermolecular induction and exchange-induction energies from coupled-perturbed Kohn-Sham density functional theory. *Chem. Phys. Lett.* **2002**, *362*, 319–325.

(S10) Hesselmann, A.; Jansen, G. First-order intermolecular interaction energies from Kohn-Sham orbitals. *Chem. Phys. Lett.* **2002**, *357*, 464–470.

(S11) Hesselmann, A.; Jansen, G. Intermolecular dispersion energies from time-dependent density functional theory. *Chem. Phys. Lett.* **2003**, *367*, 778–784.

(S12) Werner, H. J.; Knowles, P. J.; Knizia, G.; Manby, F. R.; Schutz, M. MOLPRO: a general-purpose quantum chemistry program package. *Wiley Interdiscip. Rev. Comput. Mol Sci.* **2012**, *2*, 242–253.

(S13) Werner, H. J.; Knowles, P. J.; Knizia, G.; Manby, F. R.; Schütz, M.; Celani, P.; Györffy, W.; Kats, D.; Korona, T.; Lindh, R.; Mitrushenkov, A.; Rauhut, G.; Shamasundar, K. R.; Adler, T. B.; Amos, R. D.; Bernhardsson, A.; Berning, A.; Cooper, D. L.; Deegan, M. J. O.; Dobbyn, A. J.; Eckert, F.; Goll, E.; Hampel, C.; Hesselmann, A.; Hetzer, G.; Hrenar, T.; Jansen, G.; Köppl, C.; Liu, Y.; Lloyd, A. W.; Mata, R. A.; May, A. J.; McNicholas, S. J.; Meyer, W.; Mura, M. E.; Nicklass, A.; O'Neill, D. P.; Palmieri, P.; Peng, D.; Pflüger, K.; Pitzer, R.; Reiher, M.; Shiozaki, T.; Stoll, H.; Stone, A. J.; Tarroni, R.; Thorsteinsson, T.; Wang, M. MOLPRO, version 2012.1, a package of ab initio programs. 2012; `http://www.molpro.net`.

(S14) Gruning, M.; Gritsenko, O. V.; van Gisbergen, S. J. A.; Baerends, E. J. Shape corrections to exchange-correlation potentials by gradient-regulated seamless connection of model potentials for inner and outer region. *J. Chem. Phys.* **2001**, *114*, 652–660.

(S15) Peterson, K. A.; Figgen, D.; Goll, E.; Stoll, H.; Dolg, M. Systematically convergent basis sets with relativistic pseudopotentials. II. Small-core pseudopotentials and correlation consistent basis sets for the post-d group 16-18 elements. *J. Chem. Phys.* **2003**, *119*, 11113–11123.

(S16) Lias, S. G. *NIST Chemistry WebBook,* NIST standard reference database number 69; National Institute of Standards and Technology, Gaithersburg MD, 20899, doi:10.18434/T4D303, (retrieved July 16, 2018).

(S17) Frisch, M. J.; Trucks, G. W.; Schlegel, H. B.; Scuseria, G. E.; Robb, M. A.; Cheeseman, J. R.; Scalmani, G.; Barone, V.; Petersson, G. A.; Nakatsuji, H.; Li, X.; Caricato, M.; Marenich, A. V.; Bloino, J.; Janesko, B. G.; Gomperts, R.; Mennucci, B.; Hratchian, H. P.; Ortiz, J. V.; Izmaylov, A. F.; Sonnenberg, J. L.; Williams-Young, D.; Ding, F.; Lipparini, F.; Egidi, F.; Goings, J.; Peng, B.; Petrone, A.; Henderson, T.; Ranasinghe, D.; Zakrzewski, V. G.; Gao, J.; Rega, N.; Zheng, G.; Liang, W.; Hada, M.; Ehara, M.; Toyota, K.; Fukuda, R.; Hasegawa, J.; Ishida, M.; Nakajima, T.; Honda, Y.; Kitao, O.; Nakai, H.; Vreven, T.; Throssell, K.; Montgomery, J. A., Jr.; Peralta, J. E.; Ogliaro, F.; Bearpark, M. J.; Heyd, J. J.; Brothers, E. N.; Kudin, K. N.; Staroverov, V. N.; Keith, T. A.; Kobayashi, R.; Normand, J.; Raghavachari, K.; Rendell, A. P.; Burant, J. C.; Iyengar, S. S.; Tomasi, J.; Cossi, M.; Millam, J. M.; Klene, M.; Adamo, C.; Cammi, R.; Ochterski, J. W.; Martin, R. L.; Morokuma, K.; Farkas, O.; Foresman, J. B.; Fox, D. J. Gaussian 16 Revision B.01. 2016; Gaussian Inc. Wallingford CT.

(S18) Jurecka, P.; Sponer, J.; Cerny, J.; Hobza, P. Benchmark database of accurate (MP2 and CCSD(T) complete basis set limit) interaction energies of small model complexes, DNA base pairs, and amino acid pairs. *Phys. Chem. Chem. Phys.* **2006**, *8*, 1985–1993.

(S19) Grimme, S.; Antony, J.; Ehrlich, S.; Krieg, H. A consistent and accurate ab initio

parametrization of density functional dispersion correction (DFT-D) for the 94 elements H-Pu. *J. Chem. Phys.* **2010**, *132*, 154104.

(S20) Shahbaz, M.; Szalewicz, K. Do semilocal density-functional approximations recover dispersion energies at small intermonomer separations? *Phys. Rev. Lett.* **2018**, *121*, 113402.

(S21) Verma, P.; Wang, B.; Fernandez, L. E.; Truhlar, D. G. Physical molecular mechanics method for damped dispersion. *J. Phys. Chem. A* **2017**, *121*, 2855–2862.

(S22) Podeszwa, R.; Pernal, K.; Patkowski, K.; Szalewicz, K. Extension of the Hartree-Fock plus dispersion method by first-order correlation effects. *J. Phys. Chem. Lett.* **2010**, *1*, 550–555.

(S23) Grzywa, R.; Dyguda-Kazimierowicz, E.; Sieńczyk, M.; Feliks, M.; Sokalski, W. A.; Oleksyszyn, J. The molecular basis of urokinase inhibition: From the nonempirical analysis of intermolecular interactions to the prediction of binding affinity. *J. Mol. Model.* **2007**, *13*, 677–683.

(S24) Hoja, J.; Sax, A. F.; Szalewicz, K. Is electrostatics sufficient to describe hydrogen-bonding interactions? *Chem. Eur. J.* **2014**, *20*, 2292–2300.

(S25) van der Kamp, M. W.; Chaudret, R.; Mulholland, A. J. QM/MM modelling of ketosteroid isomerase reactivity indicates that active site closure is integral to catalysis. *FEBS J.* **2013**, *280*, 3120–3131.
